# From Conventional Image Processing to Artificial Intelligence in Electron Microscopy: Toward Robust, Automated, and Enhanced Materials Characterization Through Insights from Medical and Biological Imaging

**DOI:** 10.3390/ma19214678

**Published:** 2026-11-03

**Authors:** Amanda Nieto, Leon Hamui

**Affiliations:** 1 Faculty of Engineering, Universidad Anáhuac México, Avenida Universidad Anáhuac 46, Col. Lomas Anáhuac, Huixquilucan 52786, Mexico; amanda.nieto@anahuac.mx; 2 Fidel Velázquez Technological University, Emiliano Zapata S/N, Col. El Trafico, Nicolás Romero 54400, Mexico

**Keywords:** scanning electron microscopy, transmission electron microscopy, deep learning, image restoration, image segmentation, self-supervised learning, transfer learning, multimodal learning, electron tomography, biomedical imaging

## Abstract

**Highlights:**

- Traces the evolution from conventional image processing to AI-driven electron microscopy.
- Reviews ML and DL strategies for enhancement, segmentation, reconstruction, and quantitative analysis.
- Examines self-supervised, multimodal, physics-informed, and transferable learning approaches.
- Connects advances in biomedical imaging with emerging strategies for electron microscopy.
- Identifies key challenges toward robust, automated, and generalizable materials characterization.

**Abstract:**

Electron microscopy is fundamental to modern materials characterization; however, the quantitative analysis of scanning electron microscopy (SEM) and transmission electron microscopy (TEM) data remains challenged by noise, imaging artifacts, acquisition variability, complex microstructures, and operator-dependent interpretation. This review examines the evolution of computational analysis from conventional image-processing techniques, including filtering, deconvolution, enhancement, and segmentation, toward machine learning (ML) and deep learning (DL) approaches for electron microscopy. Particular attention is given to convolutional and generative architectures, self-supervised learning, transfer learning and domain adaptation, transformers and foundation models, physics-informed learning, and multimodal integration of imaging, spectroscopic, diffraction, and three-dimensional data. Advances in medical and biological imaging are further examined to identify transferable strategies for data-efficient learning, image reconstruction, multimodal analysis, uncertainty quantification, and external validation. Collectively, these developments indicate a transition from task-specific image processing toward increasingly integrated, automated, and quantitative characterization workflows. Nevertheless, limited datasets, domain shifts across instruments and material classes, computational demands, physical consistency, and insufficient standardization remain major challenges. Future advances will require robust, generalizable, and physically informed artificial intelligence frameworks capable of integrating heterogeneous microscopy data while maintaining scientific interpretability and reliability.

## 1. Introduction

Scanning electron microscopy (SEM) and transmission electron microscopy (TEM) are indispensable tools in materials science for evaluation and characterization because of their ability to resolve micro- and nanoscale structures and to support structure–property investigations across a wide range of materials systems. SEM is widely used for surface morphology and microstructural characterization, whereas TEM enables high-resolution imaging and advanced analytical measurements, including spectroscopic modalities for compositional analysis. As materials research increasingly targets complex, multicomponent nanostructures and heterogeneous microstructures, the demand for reliable, quantitative, and reproducible microscopy-based workflows continues to grow.

SEM and TEM are routinely employed across metallurgy, semiconductor manufacturing, energy storage, catalysis, biomaterials, polymers, ceramics, geological systems, and nanotechnology. In metallic systems, electron microscopy enables the characterization of grain structures, precipitates, dislocations, and phase transformations, whereas in semiconductor devices it supports failure analysis, process monitoring, dimensional metrology, and nanoscale compositional characterization. Advanced analytical techniques such as energy-dispersive X-ray spectroscopy (EDS) and electron energy-loss spectroscopy (EELS) further extend these capabilities by providing detailed elemental and chemical information [1,2]. In nanotechnology and energy-related applications, electron microscopy enables the characterization of nanoparticles, battery electrodes, catalysts, and other nanostructured functional materials. Recent developments in three-dimensional imaging and tomographic reconstruction have further expanded these capabilities by enabling the analysis of complex microstructures and elemental distributions with high spatial resolution [3,4]. Collectively, these applications illustrate the central role of electron microscopy in connecting microstructural, compositional, and functional information across increasingly complex materials systems.

Despite these capabilities, the quantitative potential of electron microscopy remains constrained by acquisition- and specimen-dependent factors. Shot noise, detector noise, drift, blur, beam-induced damage, charging, and complex contrast mechanisms associated with electron–matter interactions can obscure relevant structural information and propagate errors into subsequent segmentation, feature detection, and compositional analysis [3,5,6,7]. These effects become particularly critical under low-dose conditions and in spectroscopic or tomographic measurements, where limited signal generation and repeated acquisitions can further compromise image quality. Understanding the physical origin and analytical consequences of these limitations is therefore essential before selecting an appropriate computational strategy for image restoration or quantitative analysis.

Traditional image-processing techniques—including filtering, deconvolution, contrast enhancement, and rule-based segmentation—have long been employed to suppress noise, compensate for blur, and support quantitative measurements in electron microscopy. These methods remain valuable because of their relatively straightforward implementation and interpretability; however, their performance frequently depends on manually selected parameters and assumptions regarding image formation and noise. Consequently, they may exhibit limited adaptability across different imaging conditions, microscope configurations, and material systems. As microstructures become increasingly heterogeneous and morphologically complex, conventional approaches may also struggle to preserve fine structural information while maintaining robust quantitative performance [5,8,9].

Beyond these analytical limitations, electron microscopy is associated with substantial capital and operational costs. SEM and TEM require specialized infrastructure, periodic maintenance, instrument calibration, and, critically, extensive involvement of highly trained personnel throughout image acquisition and interpretation. Microscope alignment, parameter optimization, feature identification, segmentation, quantitative measurement, and interpretation of complex microstructures may all require expert intervention [4,5]. This dependence on specialized operators increases both analytical variability and operational cost, particularly for materials containing overlapping phases, irregular morphologies, heterogeneous contrast distributions, or densely packed micro- and nanostructures, for which manual interpretation and segmentation become increasingly difficult [5,10,11].

At the same time, advances in detector technology and automated acquisition systems have enabled high-throughput electron microscopy, allowing increasingly large collections of high-resolution images to be generated during individual experimental campaigns. Although these developments have substantially increased acquisition capabilities, image interpretation frequently remains dependent on manual analysis, effectively shifting a major bottleneck from data acquisition to data processing and interpretation. In industrial and high-throughput research environments, this imbalance can increase characterization time and cost, restrict efficient utilization of microscopy facilities, and delay scientific or engineering decision-making [1,4].

Against this background, artificial intelligence (AI) should not be regarded merely as an alternative image-processing tool, but as a potential enabling layer for next-generation electron microscopy. Machine learning (ML) and deep learning (DL) methods can operate across multiple stages of the characterization workflow, from image restoration and feature recognition to segmentation, quantitative analysis, multimodal data integration, and, increasingly, acquisition feedback and decision support. AI-driven automation can integrate image acquisition, feature tracking, segmentation, and quantitative analysis within increasingly connected microscopy workflows, reducing operator dependence and enabling more consistent processing of large datasets [12]. This broader role is particularly relevant as electron microscopy evolves toward high-throughput, three-dimensional, and multimodal experimentation, where the volume and dimensionality of the generated data increasingly exceed the practical capacity of manual analysis. AI may therefore contribute simultaneously to analytical performance, reproducibility, throughput, instrument utilization, and operational efficiency [4,5,9,12].

Importantly, the increasing role of AI also changes the fundamental question faced by electron microscopy researchers. The challenge is no longer simply how to improve an individual image, but how to determine which computational strategy is appropriate for a particular combination of imaging physics, available data, analytical objective, and validation requirement. A method optimized for denoising may not necessarily preserve the structural information required for segmentation; a highly accurate segmentation network may fail when transferred to another microscope or material class; and a visually convincing reconstruction may not necessarily guarantee physical consistency. Thus, evaluating AI in electron microscopy requires moving beyond isolated performance improvements toward a broader consideration of robustness, transferability, interpretability, uncertainty, and integration with physically meaningful characterization objectives.

Despite the rapid expansion of AI applications in electron microscopy, the literature remains highly fragmented across individual computational tasks, imaging modalities, and learning paradigms. Image restoration, segmentation, feature detection, three-dimensional reconstruction, spectroscopic analysis, and automated acquisition are frequently addressed as separate problems, making it difficult to determine how these approaches collectively contribute to a more integrated materials-characterization workflow. Moreover, methodological advances developed in other data-intensive imaging fields—particularly medical and biological imaging—are rarely examined systematically in terms of their transferability to SEM and TEM. This fragmentation limits the ability of researchers to identify not only which algorithms are available, but also which methodological strategy is most appropriate for a given microscopy challenge.

Accordingly, the distinctive contribution of this review is not simply to catalogue ML and DL architectures applied to electron microscopy, but to organize the field around the analytical problems that must be solved to achieve robust, quantitative, and increasingly autonomous materials characterization. The review connects image-formation constraints and conventional processing strategies with classical and modern ML, deep convolutional and generative architectures, self-supervised learning, transfer learning and domain adaptation, transformers and foundation models, physics-informed learning, and multimodal approaches. These methods are examined in relation to recurring microscopy requirements, including noise and artifact mitigation, structural preservation, segmentation, three-dimensional reconstruction, compositional integration, uncertainty assessment, generalization across instruments and material classes, and reduction of operator dependence.

From a practical perspective, this organization is intended to provide researchers with a decision-oriented framework for navigating the rapidly expanding AI landscape in electron microscopy. Rather than beginning with a particular algorithm, researchers can begin with an experimental or analytical limitation—such as low signal-to-noise ratio, limited annotated data, complex morphology, domain shift between instruments, multimodal measurements, or the need for three-dimensional reconstruction—and identify the corresponding families of computational strategies, their principal advantages and limitations, and the emerging approaches that may overcome those constraints. In this sense, the review is intended not only as a synthesis of the state of the art but also as a methodological roadmap connecting microscopy challenges, computational solutions, validation requirements, and future opportunities for intelligent materials characterization.

A further distinctive element of this framework is the cross-disciplinary analysis of medical and biological imaging. These fields have confronted several challenges that parallel those now becoming increasingly relevant in electron microscopy, including noisy and heterogeneous data, limited annotations, instrument- and institution-dependent domain shifts, three-dimensional reconstruction, multimodal integration, uncertainty quantification, and the requirement for robust external validation. Examining how these challenges have been addressed in biomedical imaging can therefore reveal transferable methodological principles for developing more data-efficient, generalizable, and trustworthy AI systems for electron microscopy. Importantly, this cross-disciplinary exchange can also operate in the opposite direction. Advances in AI-assisted electron microscopy—including high-resolution image enhancement, automated segmentation, three-dimensional reconstruction, and the integration of morphological, compositional, and spectroscopic information—can support the characterization of cells, tissues, biomaterials, and disease-related ultrastructural changes at micro- and nanometer scales. The convergence of these approaches may therefore accelerate the identification of biologically relevant structural and molecular features, facilitate the development and evaluation of biomaterials and medical technologies, and ultimately contribute to more efficient investigation of biomedical problems. The objective is not to imply direct equivalence between biomedical and materials imaging, which rely on fundamentally different physical mechanisms and analytical objectives, but rather to identify computational principles that can be transferred, adapted, and physically constrained for materials characterization.

On this basis, this review critically examines the transition from conventional image processing toward increasingly intelligent electron microscopy workflows. The discussion progresses from the physical origins of image formation, noise, and artifacts in SEM and TEM to conventional restoration and segmentation techniques, followed by classical ML and contemporary DL approaches for image enhancement, segmentation, reconstruction, multimodal analysis, and quantitative characterization. Particular attention is given to emerging learning strategies that improve data efficiency, generalization, physical consistency, and the integration of complementary microscopy information.

These developments are subsequently examined alongside advances in medical and biological imaging to identify transferable methodological principles and validation strategies. Through this structure, the review seeks to clarify not only how AI is currently being used in electron microscopy, but also how complementary computational approaches can enable more efficient, robust, and reproducible characterization workflows. In this context, we use the term smart characterization to describe the progression toward integrated frameworks in which AI supports the acquisition, processing, multimodal integration, quantitative interpretation, and validation of microscopy data, while adapting analytical decisions to the information and uncertainty associated with each experiment. Such a framework may ultimately connect materials characterization and biomedical research more effectively by accelerating the extraction of reliable information from increasingly complex imaging and spectroscopic datasets.

## 2. Image Formation and Challenges in SEM and TEM

SEM and TEM rely on fundamentally different electron–matter interaction mechanisms, which determine the origin of image contrast, the information encoded in the detected signal, and the artifacts that may arise during acquisition. Understanding these physical mechanisms is essential for both conventional image processing and AI-based analysis because computational models operate on image intensities whose meaning is intrinsically linked to signal generation and detection. Consequently, reliable computational workflows must distinguish meaningful microstructural information from variations introduced by acquisition conditions, detector response, specimen characteristics, and imaging physics.

In SEM, image formation is primarily based on the detection of secondary electrons (SEs) and, in some configurations, backscattered electrons (BSEs) emitted from the specimen following interaction with the incident electron beam. SE imaging is particularly sensitive to surface morphology and topography, whereas BSE intensity is strongly influenced by atomic number and therefore provides useful compositional contrast. However, the measured intensity is also affected by factors such as surface orientation, electron landing energy, detector geometry, and collection efficiency. Consequently, intensity variations in SEM images cannot always be interpreted exclusively as differences in material composition or microstructure [5,13].

TEM, by contrast, forms images using electrons transmitted through an electron-transparent specimen, with contrast arising from elastic and inelastic scattering processes. Different imaging configurations, including bright-field TEM, dark-field TEM, and high-angle annular dark-field scanning TEM (HAADF-STEM), provide complementary information related to crystallography, mass–thickness contrast, and atomic-number contrast [1,14]. Although TEM provides substantially higher spatial resolution than conventional SEM, the interaction of high-energy electrons with thin specimens can also produce beam-induced structural or chemical modifications, particularly in beam-sensitive materials and during high-dose analytical measurements.

These differences in image formation have direct implications for computational analysis. Similar intensity patterns may originate from different physical phenomena depending on the microscopy mode, specimen, detector, or acquisition conditions, while the same microstructural feature may exhibit substantially different contrast under different imaging configurations. Algorithms developed for enhancement, segmentation, classification, or reconstruction therefore cannot be assumed to operate independently of the physical origin of the input data. This issue becomes particularly relevant for learning-based methods, which may otherwise learn correlations associated with a specific microscope configuration, acquisition protocol, or specimen class rather than physically meaningful characteristics of the material.

Advanced electron microscopy workflows further increase this complexity by combining imaging with spectroscopic and three-dimensional information. SEM and TEM are frequently coupled with energy-dispersive X-ray spectroscopy (EDS) and electron energy-loss spectroscopy (EELS) to obtain elemental and chemical information. Although these modalities provide valuable compositional insights, they are affected by relatively low signal-generation probabilities, which can result in noisy datasets and longer acquisition times [2,3,14]. Similarly, three-dimensional (3D) tomographic reconstruction requires multiple projections, increasing both experimental duration and cumulative electron exposure [6].

These considerations reveal a fundamental trade-off among spatial resolution, signal quality, acquisition time, and specimen integrity. Increasing electron dose can improve signal statistics but may induce beam damage, whereas reducing dose can preserve the specimen at the expense of image quality. Longer acquisition times may improve signal quality but increase susceptibility to drift and reduce experimental throughput. Furthermore, specimen preparation, detector performance, environmental stability, and the underlying physics of electron–matter interactions impose constraints that cannot necessarily be eliminated through experimental optimization alone [15]. These limitations become increasingly relevant in modern high-resolution and high-throughput workflows, where greater spatial resolution and measurement sensitivity may be achieved at the expense of acquisition time, specimen stability, or experimental throughput.

Specimen preparation represents an additional source of uncertainty in quantitative electron microscopy, as preparation-induced deformation, contamination, thickness variations, aggregation, and sampling bias may affect the structural information recorded in SEM and (S)TEM images [16]. These limitations are particularly relevant for beam-sensitive materials, including metal–organic frameworks (MOFs), zeolites, polymeric nanostructures, and biological specimens, for which electron irradiation may induce structural changes and necessitate low-dose acquisition [17,18]. Deep-learning-based denoising has demonstrated potential for improving the interpretability of low-dose cryo-electron microscopy images and tomograms, as illustrated by Topaz-Denoise [19], while related self-supervised approaches have also been investigated for atomic-resolution STEM imaging [20]. Nevertheless, the applicability of these methods must be evaluated for each specimen class and imaging modality, since computational enhancement cannot reverse preparation-induced structural changes or guarantee the recovery of information that was not reliably recorded. Consequently, reliable AI-assisted characterization requires representative specimen preparation, appropriate acquisition protocols, and independent validation of reconstructed or segmented features.

These considerations also extend to the construction of datasets for machine learning. Differences in specimen preparation, including thickness, surface condition, contamination, aggregation, and preparation-induced damage, can introduce systematic variations that influence both image appearance and the annotations used for model training. Consequently, datasets should document relevant preparation procedures and, where possible, include representative variations across specimens and preparation conditions. Without such diversity, a model may learn preparation-specific features rather than generalizable microstructural characteristics, limiting the reliability of subsequent quantitative analysis.

Computational methods therefore play a complementary rather than purely corrective role in modern electron microscopy. Image restoration, noise suppression, super-resolution, segmentation, and quantitative feature extraction can potentially increase the amount of useful information obtained from experimental data while reducing the need for repeated acquisitions or excessive electron exposure [4,8]. Importantly, however, the reliability of these approaches ultimately depends on whether physically meaningful information is preserved rather than merely producing visually improved images. This distinction provides an important criterion for evaluating AI-based electron microscopy: computational performance should be assessed not only in terms of visual appearance or numerical reconstruction accuracy, but also according to the preservation of structural and compositional information required for subsequent scientific interpretation.

**Figure 1:**
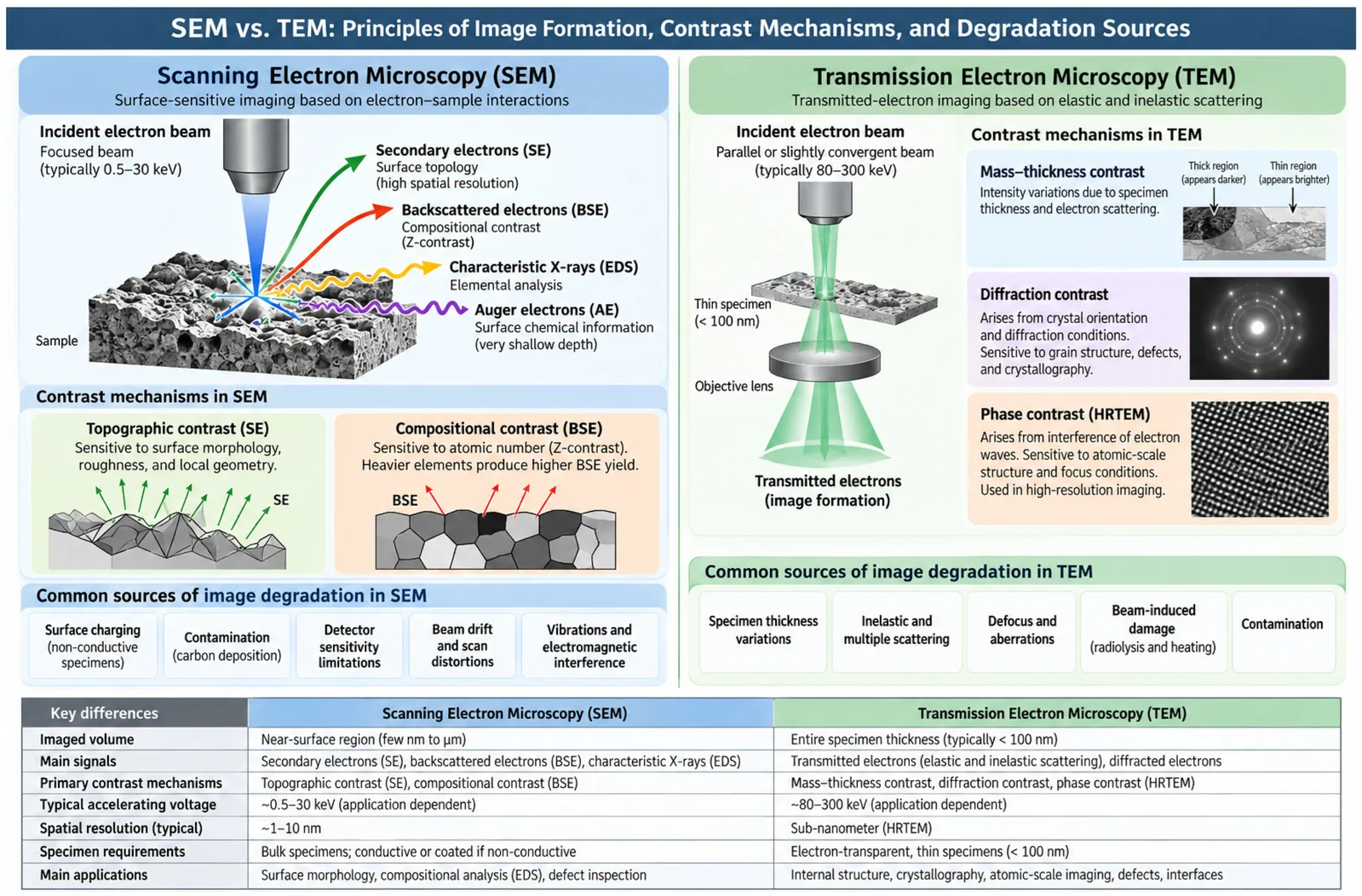
Comparative schematic of image formation, contrast mechanisms, and common sources of image degradation in scanning electron microscopy (SEM) and transmission electron microscopy (TEM). The SEM panel illustrates electron–sample interactions, secondary electron (SE) and backscattered electron (BSE) signals, characteristic X-ray emission for energy-dispersive X-ray spectroscopy (EDS), and the principal mechanisms of topographic and compositional contrast. The TEM panel illustrates transmitted-electron imaging and distinguishes mass–thickness, diffraction, and phase contrast, depending on the imaging mode. Representative sources of image degradation are summarized for both techniques. The schematic is based on the electron microscopy principles described by Goldstein et al. [21] and Reimer and Kohl [22]. The figure was developed by the authors with the assistance of Google Gemini. All microscopy-like visual elements are conceptual illustrations and do not represent experimental micrographs or measured data.

Although SEM and TEM are both based on electron–matter interactions, their signal-generation mechanisms, detection geometries, and contrast-formation processes differ substantially. These differences determine not only the structural and compositional information available to the researcher but also the types of artifacts and acquisition-related variations that computational methods must address. Figure 1 summarizes the principal image-formation pathways in SEM and TEM, together with their characteristic detection mechanisms, contrast sources, and representative factors that may compromise image quality.

Consequently, image quality in electron microscopy reflects the combined influence of electron–sample interactions, acquisition conditions, detector response, specimen characteristics, and environmental stability. Establishing this physical context is essential before evaluating image-processing or learning-based methods because an algorithm can only be considered scientifically reliable when the information it restores, segments, or quantifies remains consistent with the underlying microscopy signal.

### Noise Sources and Signal-to-Noise Ratio in Electron Microscopy

Among the acquisition-related factors discussed above, noise and signal variability deserve particular attention because they directly determine which microstructural information remains detectable and how reliably that information can subsequently be quantified. This issue is especially relevant for AI-based analysis: noise can obscure physically meaningful features, introduce spurious intensity patterns, and modify the statistical distribution of the data used for model training and inference. Understanding the origin and behavior of noise is therefore necessary not only for selecting appropriate preprocessing strategies, but also for determining whether an apparent improvement produced by a learning-based model corresponds to genuine information recovery or to algorithmically generated image content.

The signal-to-noise ratio (SNR) provides a useful measure of the relationship between the information-carrying signal and unwanted fluctuations introduced during acquisition. Higher SNR generally improves the visibility of microstructural features and increases confidence in subsequent quantitative measurements. However, SNR in electron microscopy is determined by several interconnected physical and instrumental factors and cannot be considered independently of electron dose, detector response, acquisition conditions, and specimen sensitivity.

Electron microscopy images are formed through interactions between the incident electron beam and the specimen, generating detectable signals with distinct physical origins. In SEM, SEs and BSEs provide complementary topographical and compositional information, respectively [21,22]. Nevertheless, the stochastic nature of electron detection introduces statistical fluctuations into both signals. Their measured intensity also depends on atomic number, surface topography, crystallographic orientation, beam energy, detector geometry, and collection efficiency. Variations in these factors can generate intensity fluctuations unrelated to the microstructural feature of interest, thereby reducing contrast and complicating quantitative image analysis. Contamination, detector sensitivity, and beam drift can further alter the detected signals and adversely affect segmentation, feature extraction, and subsequent machine-learning analyses [5,21].

One of the dominant sources of noise affecting electron microscopy is shot noise, which originates from the discrete nature of electron detection. Because electrons are detected as individual events, the measured signal follows Poisson statistics. These statistical fluctuations become increasingly significant as electron dose decreases: fewer detected electrons increase the relative contribution of shot noise, reducing SNR and making the reliable identification of fine structural features more difficult [6]. This represents a particularly important constraint for beam-sensitive specimens, for which increasing electron dose to improve signal statistics may not be experimentally acceptable.

Additional noise and instability can originate from detector electronics, signal-amplification circuitry, mechanical vibration, electromagnetic interference, and thermal fluctuations. Electronic noise may introduce random intensity variations, whereas mechanical and electromagnetic disturbances can produce distortions or spatial instability. Thermal fluctuations within detector systems may further affect signal stability, particularly during extended acquisition periods. In SEM, charging of non-conductive or poorly conductive specimens can additionally modify local electric fields and generate contrast variations, image displacement, or unstable signal intensity. Complex surface topography may also produce shadowing and orientation-dependent contrast that complicate automated image analysis [5].

In TEM and STEM, beam-induced specimen damage represents an additional constraint. High-energy electron beams may alter the structure, chemistry, or morphology of sensitive materials during observation. Low-dose acquisition strategies are therefore frequently employed to preserve specimen integrity; however, reducing electron exposure inevitably decreases the number of detected electrons and consequently the SNR [4]. This produces a fundamental compromise between the amount of information obtained from an image and preservation of the original specimen.

From a computational perspective, these effects are particularly consequential because multiple sources of noise and acquisition variability may coexist within the same dataset. Their combined influence can obscure low-contrast features, reduce boundary definition, modify local intensity distributions, and decrease the separability of adjacent particles, phases, grains, or defects. Image restoration therefore constitutes an inverse problem in which the computational method must distinguish information generated by the specimen from variations introduced during acquisition. Conventional filtering and deconvolution can partially address these effects, whereas learning-based approaches can model more complex statistical relationships within experimental data [8,9]. Greater modeling capacity, however, also introduces an important validation requirement: restored or reconstructed features must correspond to experimentally supported information rather than artificial structures generated by the computational model.

**Figure 2:**
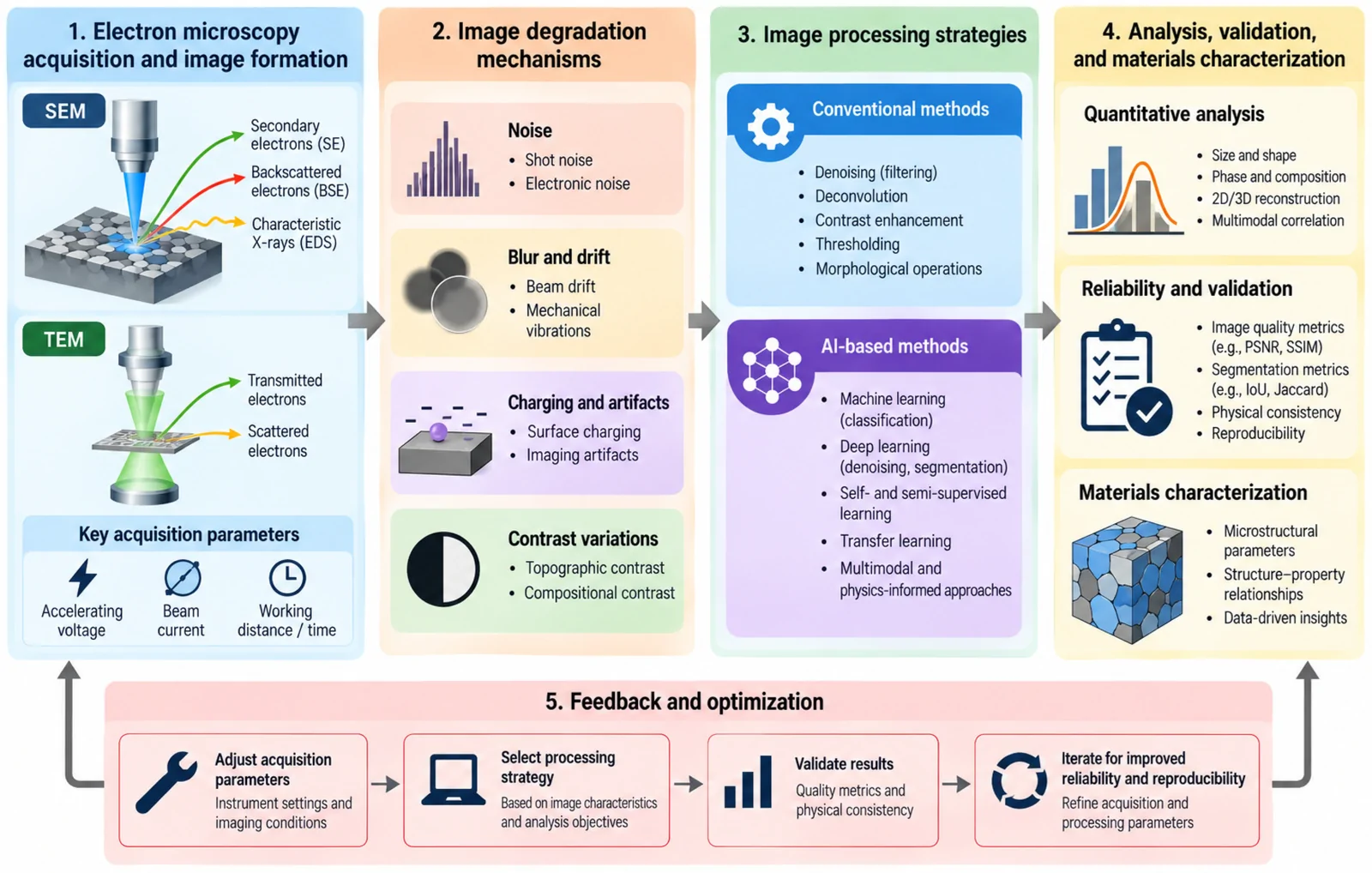
Conceptual methodological framework connecting electron microscopy image formation, image degradation, computational processing, and reliable materials characterization. The diagram summarizes SEM and TEM acquisition principles; representative degradation mechanisms, including noise, blur, drift, charging, and contrast variations; conventional and AI-based image-processing strategies; and subsequent quantitative analysis and validation. The feedback loop illustrates the iterative adjustment of acquisition and processing parameters to improve reliability and reproducibility. The diagram summarizes general relationships relevant to SEM and TEM imaging and does not represent experimental micrographs or results from a specific material system. Schematic developed by the authors using Microsoft PowerPoint. No generative AI tools were used in its preparation.

The combined influence of noise, blur, beam drift, charging, and contrast variations extends beyond visual image quality and can propagate into downstream analysis. These acquisition-related limitations may reduce feature detectability, alter apparent boundaries, and compromise segmentation and quantitative measurements across different material systems. Figure 2 presents a conceptual methodological framework linking SEM and TEM image formation, common degradation mechanisms, and their consequences for subsequent computational analysis. The diagram illustrates how conventional image-processing and AI-based strategies can address these challenges, while emphasizing the importance of quantitative validation, physical consistency, and iterative optimization for reliable materials characterization.

Taken together, the image-formation principles and noise mechanisms discussed in Section 2 and its subsection “Noise Sources and Signal-to-Noise Ratio in Electron Microscopy”, demonstrate that the central challenge in electron microscopy image analysis is not simply the presence of noise or artifacts, but the close coupling among imaging physics, acquisition conditions, specimen response, and downstream computational interpretation. Noise, drift, charging, dose limitations, detector response, and contrast mechanisms can alter both the visibility and the statistical representation of microstructural features, allowing errors introduced during acquisition to propagate into segmentation, reconstruction, classification, and quantitative measurements. This has an important implication for AI-assisted microscopy: successful models must do more than improve apparent image quality; they must preserve physically and scientifically meaningful information and remain robust to variations in acquisition conditions. Image-formation knowledge should therefore be considered part of the computational problem itself rather than an external experimental consideration.

The following section therefore examines the traditional image-processing techniques that have been widely employed to address these challenges in SEM and TEM images.

## 3. Traditional Image Processing Techniques for SEM and TEM Images

Prior to the widespread adoption of machine-learning-based approaches, enhancement and quantitative analysis of SEM and TEM images relied predominantly on traditional image-processing techniques grounded in signal-processing theory and mathematical models. These methods remain widely used because they are computationally accessible, relatively interpretable, and effective when the characteristics of the noise, blur, contrast distribution, or objects of interest are sufficiently well defined.

Traditional processing strategies address different stages of electron microscopy analysis. Filtering methods primarily suppress unwanted intensity fluctuations; deconvolution attempts to recover spatial information affected by the imaging system; and thresholding and morphological operations transform image intensity into discrete regions that can subsequently be quantified. These approaches therefore represent more than historical predecessors of artificial intelligence: they establish many of the fundamental operations, assumptions, and validation criteria against which learning-based methods continue to be evaluated.

**Figure 3:**
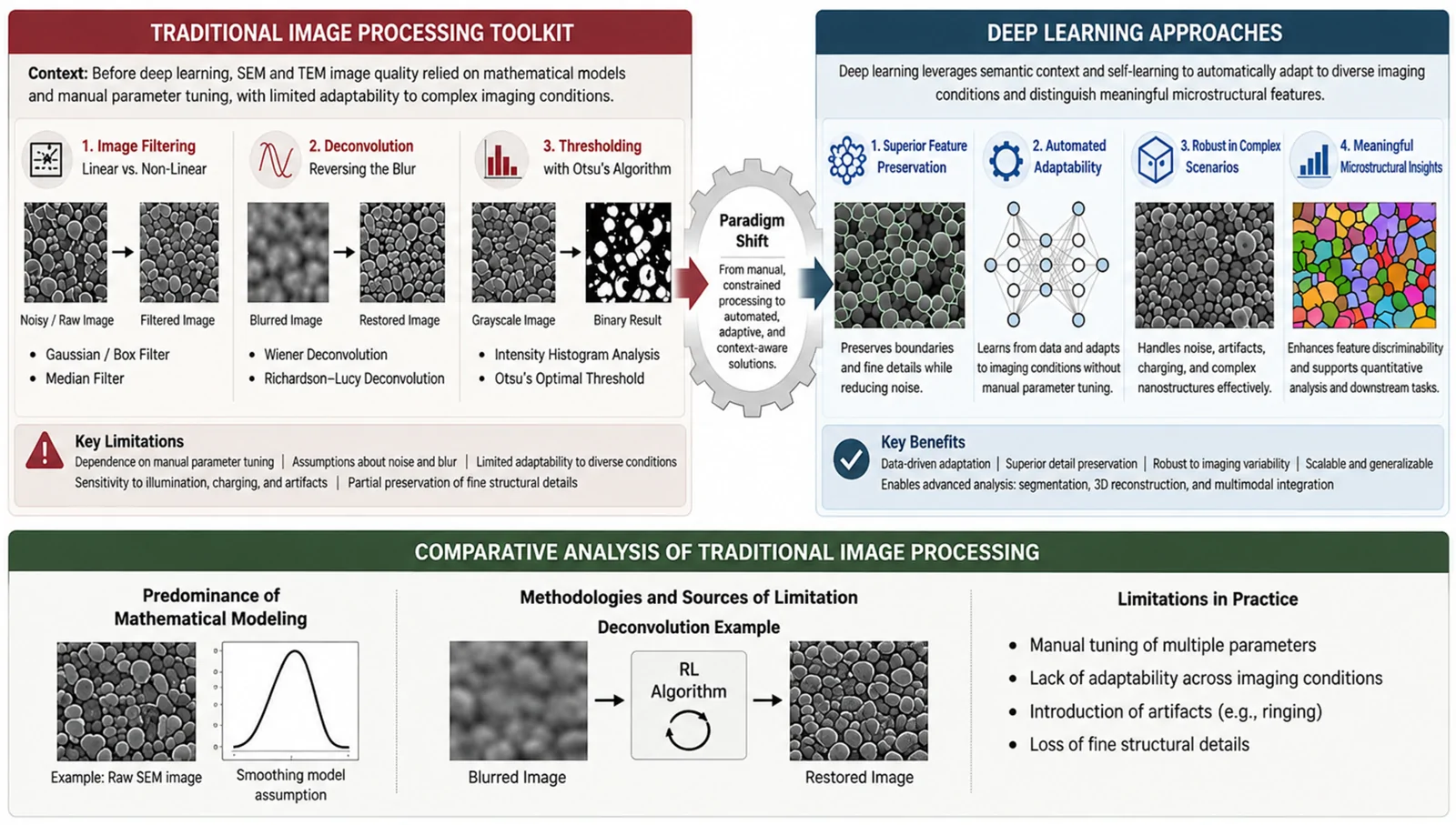
Conceptual comparison of conventional and deep-learning-based approaches for SEM/TEM image processing. Conventional methods—including filtering, deconvolution, and threshold-based segmentation—provide established strategies for noise reduction, image restoration, and feature extraction, although their performance may depend on predefined mathematical assumptions and parameter selection. Deep-learning approaches offer data-driven alternatives capable of learning complex image features and adapting to different imaging conditions, but their reliability depends on representative training data and appropriate validation. The lower panel illustrates the mathematical assumptions, restoration principles, and practical limitations associated with conventional processing techniques. All microscopy-like images and processing outcomes are schematic representations rather than experimental results or quantitative benchmarks. Abbreviations: RL, Richardson–Lucy deconvolution; PSNR, peak signal-to-noise ratio; SSIM, structural similarity index measure. Figure created by the authors using Google Gemini.

Their performance, however, depends strongly on the relationship between the mathematical assumptions of the method and the physical characteristics of the acquired data. Parameter selection, noise statistics, point-spread-function estimation, local contrast, and object morphology can substantially influence the final result. Figure 3 provides an integrated overview of these conventional strategies, their principal limitations, and their conceptual relationship with data-driven approaches.

As illustrated in Figure 3, conventional techniques can provide substantial improvements when the characteristics of the imaging problem are sufficiently well defined. Filtering can suppress unwanted intensity fluctuations, deconvolution can partially recover spatial information affected by blur, and thresholding provides a computationally efficient means of separating features according to intensity distributions. Nevertheless, these operations involve trade-offs among noise suppression, edge preservation, artifact generation, computational requirements, and sensitivity to parameter selection.

Objective assessment is therefore necessary to determine whether an apparent visual improvement corresponds to meaningful preservation of microstructural information. Peak Signal-to-Noise Ratio (PSNR) is one of the most widely used metrics for restoration tasks and quantifies reconstruction error relative to the maximum possible image intensity, typically in decibels [23]. Higher PSNR values generally indicate lower numerical distortion; however, because PSNR is based on pixel-wise error, it does not explicitly account for structural information. Images with similar PSNR values may therefore preserve substantially different levels of particle-boundary, grain, or phase-interface information, which limits the use of PSNR as an isolated indicator of microscopy-image quality [23].

The Structural Similarity Index Measure (SSIM) provides a complementary assessment by comparing luminance, contrast, and structural information [24]. Values approaching unity indicate greater structural similarity to the reference image. In electron microscopy, this structural sensitivity is particularly relevant because preservation of morphology may be more important for subsequent quantitative analysis than minimization of pixel-wise error alone. Studies involving particle characterization and learning-based image enhancement have consequently used PSNR and SSIM together to evaluate numerical reconstruction accuracy and structural fidelity [10,13].

PSNR and SSIM should be interpreted as complementary indicators rather than as evidence that a single numerical metric defines image quality. Conventional methods may improve numerical reconstruction or structural similarity under well-characterized conditions, whereas learning-based restoration can potentially model more complex relationships between the input data and the underlying image structure. However, the performance of either approach depends on the imaging conditions, data characteristics, and evaluation criteria. Importantly, higher metric values do not by themselves establish physical fidelity; quantitative evaluation should remain linked to the preservation of microstructural information required for the intended analytical task.

The following subsections examine these traditional approaches according to their principal analytical function. Section 3.1 and Section 3.2 first consider filtering and model-based restoration, which primarily address unwanted signal variation and loss of spatial information, respectively. Section 3.3 then examines thresholding and rule-based segmentation for converting processed images into quantitatively measurable microstructural regions. Their collective limitations and the implications for subsequent data-driven analysis are discussed in Section 3.4 and Section 3.5.

### 3.1. Image Filtering Techniques

Filtering techniques constitute one of the most established approaches for improving electron microscopy images. Linear filters, such as Gaussian and box filters, reduce high-frequency intensity fluctuations by averaging pixel values within a local neighborhood [25]. Gaussian filtering can effectively suppress random noise, but this improvement may be accompanied by blurring of edges and fine structural details that are important for accurate microstructural characterization.

Non-linear filters, including median filters, provide an alternative when preservation of boundaries is particularly important. Median filtering is effective for removing impulse-like noise while generally preserving edges better than linear averaging filters [26]. Nevertheless, SEM and TEM images frequently contain heterogeneous contrast, texture-dependent features, and structures spanning multiple spatial scales. Under these conditions, even non-linear filtering may distort morphological boundaries or remove subtle features relevant to subsequent quantitative measurements.

The effectiveness of a filter therefore depends strongly on the statistical characteristics of the noise and on the spatial scale of the features that must be preserved. Comparative studies indicate that no single filtering strategy consistently performs best across different image types and noise conditions [25]. This dependence on image-specific parameter selection limits the direct transfer of fixed filtering pipelines to heterogeneous or high-throughput microscopy datasets.

Thus, the principal value of conventional filtering lies in its simplicity, interpretability, and effectiveness under well-characterized noise conditions, whereas its principal limitation is the inherent trade-off between suppressing noise-related intensity fluctuations and preserving fine microstructural features, such as particle boundaries, interfaces, defects, and local texture.

### 3.2. Deconvolution and Model-Based Restoration

Whereas filtering primarily suppresses unwanted signal variation, deconvolution seeks to recover spatial information lost through the imaging process. The observed image is typically modeled as a convolution between the underlying object and a point spread function (PSF), together with a noise contribution. Restoration then attempts to invert this relationship.

In electron microscopy, deconvolution has been employed to compensate for instrumental blur, beam broadening, and detector-related effects [9]. Common approaches include Wiener filtering, Richardson–Lucy deconvolution, and regularized inverse methods. When the PSF and noise characteristics are adequately estimated, these techniques can improve image sharpness and recover spatial detail.

Their effectiveness, however, depends strongly on the validity of the assumed image-formation model. In practical SEM and TEM experiments, the PSF may vary spatially and can be difficult to estimate because of electron–matter interactions, specimen-dependent contrast, charging, and instrumental conditions [5]. Iterative deconvolution methods may also amplify noise or introduce ringing and over-sharpening artifacts, particularly in low-SNR images. These effects become especially relevant in low-dose experiments, where electron exposure must be limited to preserve specimen integrity [6].

Model-based restoration therefore illustrates a central trade-off of traditional image processing: incorporating physical assumptions can make the reconstruction process interpretable, but inaccuracies in those assumptions can propagate directly into the restored image. This dependence on explicit models becomes increasingly restrictive when acquisition conditions are heterogeneous or difficult to characterize.

### 3.3. Thresholding and Rule-Based Segmentation

For quantitative microstructural characterization, image enhancement is frequently followed by segmentation to isolate particles, grains, phases, pores, defects, or other regions of interest. Traditional segmentation pipelines commonly use intensity thresholding, often combined with morphological operations.

Global methods such as Otsu’s algorithm determine an intensity threshold from the image histogram and can be effective when the structures of interest produce clearly separated intensity distributions. Electron microscopy images, however, frequently violate this assumption because signal intensity can vary with surface morphology, specimen orientation, local composition, charging, illumination, and detector geometry [5]. A single global threshold may therefore merge distinct structures or incorrectly separate regions belonging to the same phase.

Rule-based strategies can extend thresholding through morphological operations such as erosion, dilation, opening, closing, and watershed segmentation. These methods are useful for removing small artifacts, refining object boundaries, and separating touching particles. They can perform effectively for relatively well-defined structures but become less reliable for irregular morphologies, overlapping particles, heterogeneous phases, or densely packed nanoparticle assemblies [10,11].

The main limitation of traditional segmentation is therefore not simply the thresholding operation itself, but its dependence on predefined intensity and geometric rules. As microstructures become more heterogeneous, the relationship between pixel intensity and physical class becomes increasingly ambiguous, reducing the robustness of fixed rule-based pipelines.

### 3.4. Limitations of Traditional Approaches

Traditional image-processing methods remain valuable components of electron microscopy workflows and should not be interpreted as obsolete simply because learning-based alternatives are increasingly available. Their transparency, low computational cost, limited data requirements, and direct relationship with established signal-processing principles make them particularly useful when acquisition conditions are controlled, and the analytical objective is well defined.

Their limitations become more apparent as the complexity and variability of the data increase. First, filtering, deconvolution, and rule-based segmentation frequently require manual parameter selection, introducing operator dependence and reducing reproducibility across datasets. Second, parameters optimized for one microscope, detector configuration, material system, magnification, or acquisition condition may not transfer reliably to another. Third, many conventional methods operate primarily on local intensities or predefined mathematical relationships and therefore have limited ability to incorporate higher-level contextual information when distinguishing relevant microstructural features from artifacts or background variation.

These constraints are especially important for images containing overlapping particles, heterogeneous phases, irregular morphologies, low-contrast interfaces, or multimodal information [8]. In such cases, a sequence of individually reasonable processing operations can accumulate errors across the workflow. For example, excessive smoothing can alter a boundary before thresholding, and inaccurate segmentation can subsequently bias particle-size distributions or phase-fraction measurements. The performance of the complete analytical pipeline is therefore determined not only by the effectiveness of each individual operation but also by how errors propagate between processing stages.

Taken together, traditional methods are most effective when the relationship between image characteristics and the desired analytical output can be described explicitly. Their decreasing robustness as that relationship becomes heterogeneous, context-dependent, or difficult to model represents one of the principal motivations for data-driven approaches. Importantly, this transition should not be interpreted as a simple replacement of conventional processing by AI; rather, it reflects a shift from explicitly programmed assumptions toward representations learned from data, while retaining the need for physical interpretation and rigorous validation.

### 3.5. Evaluation and Benchmarking for Reliable Electron Microscopy Image Analysis

The transition from conventional image processing to learning-based methods also requires appropriate criteria for determining whether a computational result represents a genuine analytical improvement. Image quality cannot be evaluated exclusively by visual appearance, particularly in electron microscopy, where subtle modifications to particle boundaries, grain structures, phase interfaces, or defects can affect subsequent quantitative characterization.

As discussed above, PSNR and SSIM provide complementary information for restoration and enhancement tasks. PSNR primarily reflects numerical reconstruction error, whereas SSIM provides greater sensitivity to structural similarity [23,24]. Their combined use is therefore preferable to reliance on either metric alone. Nevertheless, both metrics have limitations: they typically require an appropriate reference image and do not directly determine whether a reconstructed feature is physically correct or whether it will support accurate downstream measurements.

For segmentation tasks, evaluation must instead quantify agreement between predicted and reference regions. Precision, recall, F1-score, the Dice Similarity Coefficient (DSC), and Intersection over Union (IoU) are commonly employed for this purpose. The Dice coefficient [27] and IoU, derived from the Jaccard index [28], quantify spatial overlap between predicted and reference regions and have been widely used for particle segmentation, phase identification, and defect-detection applications in electron microscopy [11,29].

Metric selection should therefore be aligned with the analytical objective. Restoration algorithms require assessment of image fidelity and structural preservation, whereas segmentation models require measures of spatial agreement and classification performance. For quantitative materials characterization, an additional level of validation may be necessary: the processed output should preserve the physical measurements ultimately derived from the image, such as particle size, morphology, phase fraction, boundary location, or defect density. This task-oriented perspective is particularly important when comparing traditional and learning-based approaches because an improvement in PSNR or SSIM does not necessarily guarantee improvement in the final scientific measurement.

Table 1 compares representative conventional image-processing techniques and deep-learning-based approaches for SEM and TEM image enhancement and restoration, highlighting their principal strengths, limitations, and indicative PSNR and SSIM trends. The methods are grouped according to their underlying computational principles to distinguish model-based and deterministic processing from data-driven learning approaches. The reported trends should be interpreted qualitatively, since performance depends on the dataset, noise characteristics, reference-image quality, acquisition conditions, and implementation parameters.

Standardized evaluation therefore represents more than a reporting requirement; it is necessary for determining whether apparent algorithmic improvements translate into reproducible scientific information. The use of complementary image-quality, segmentation, and task-specific metrics, together with representative datasets and clearly defined acquisition conditions, provides a more meaningful basis for comparison than any single metric alone. As microscopy datasets become more heterogeneous, standardized benchmarking and publicly available reference datasets will become increasingly important for assessing robustness and enabling objective comparisons across computational approaches [5].

Conventional image-processing techniques continue to play an essential role in electron microscopy because they offer practical advantages that are not necessarily matched by learning-based approaches. Gaussian and median filtering, Wiener and Richardson–Lucy deconvolution, contrast-limited adaptive histogram equalization (CLAHE), and rule-based segmentation are generally interpretable, relatively straightforward to implement, and do not require extensive training datasets. Their computational efficiency and explicit parameterization make them particularly useful for exploratory characterization, routine analysis, and applications involving limited experimental data. When image degradation is sufficiently characterized, and the analytical task is well defined, conventional methods can provide reliable results without the additional complexity of deep neural networks. For example, Karabağ et al. [30] compared a traditional image-processing pipeline with four deep-learning architectures for nuclear-envelope segmentation in serial block-face scanning electron microscopy images and reported superior segmentation performance for the conventional approach in their evaluated dataset. This example illustrates that algorithmic complexity does not necessarily translate into greater quantitative accuracy and that method selection should remain application-dependent.

Importantly, conventional image-processing techniques are not restricted to standalone applications but also provide complementary tools within machine-learning and deep-learning workflows. Filtering, background correction, contrast normalization, and morphological operations can support image standardization, facilitate the identification of candidate regions of interest, and contribute to the preparation of training datasets. Traditional thresholding, edge detection, and watershed-based segmentation may also be used to generate preliminary masks or assist manual annotation, potentially reducing the effort required for supervised learning. Hybrid workflows can therefore combine conventional preprocessing with learning-based segmentation, classification, or restoration. However, these operations must be selected carefully, since excessive smoothing or contrast enhancement may suppress weak but physically meaningful features, modify apparent boundaries, or introduce systematic distortions. Similarly, automatically generated masks should not be considered validated ground truth without expert inspection or independent verification, because annotation errors and preprocessing artifacts may be propagated into model training [31].

Despite their flexibility, deep-learning approaches introduce limitations that should be considered alongside their demonstrated advantages. Model performance depends on the quality, diversity, and representativeness of training data, which may not adequately capture differences in specimen composition, morphology, preparation, microscope configuration, detector response, or acquisition conditions. Consequently, models trained on restricted datasets may exhibit reduced reliability when applied to previously unseen instruments, imaging modalities, or material systems. Supervised methods may require substantial annotation effort, whereas self-supervised approaches remain dependent on assumptions regarding noise statistics, data independence, or image structure. Furthermore, learning-based restoration and generative reconstruction can produce visually plausible details that are insufficiently supported by the measured signal, potentially compromising subsequent segmentation and quantitative characterization. Such risks have been discussed extensively in microscopy-oriented assessments of deep learning [31,32]. Accordingly, improvements in apparent image quality or numerical reconstruction metrics should not be interpreted as definitive evidence of improved scientific accuracy without independent structural or task-specific validation.

Computational cost represents another important consideration when comparing conventional and learning-based approaches. Traditional filtering, contrast enhancement, and thresholding can often be implemented efficiently on standard computing hardware, although iterative deconvolution and large multidimensional datasets may also require substantial processing resources. Deep-learning workflows, by contrast, may involve computationally intensive model training, hyperparameter optimization, repeated validation, and considerable memory requirements, particularly for high-resolution electron microscopy images, volumetric datasets, and three-dimensional reconstruction tasks. Nevertheless, computational comparisons must distinguish between training and inference: once trained, a neural network may process new images efficiently, potentially distributing its initial development cost across large datasets or repeated analyses. Actual computational efficiency therefore depends on image dimensions, network architecture, implementation, hardware, training frequency, and processing throughput. Rather than assuming the universal superiority of either methodological family, a balanced evaluation should consider segmentation or restoration accuracy, computational requirements, interpretability, robustness, and preservation of physically meaningful information. Conventional image processing and learning-based methods should therefore be considered complementary rather than mutually exclusive. In practical terms, the choice between filtering, restoration, and segmentation approaches depends on the characteristics of the acquired images and the intended quantitative analysis. The examples summarized in Table 1 and Figure 3 illustrate these trade-offs, including the simplicity and interpretability of conventional techniques, the adaptability of learning-based methods, and the importance of evaluating their limitations using experimentally meaningful criteria.

## 4. Machine Learning and Deep Learning Approaches for SEM and TEM Image Analysis

Building on the limitations and validation requirements identified for conventional image-processing methods, ML and DL introduce a fundamentally different computational strategy for SEM and TEM analysis: rather than relying exclusively on predefined transformations or manually engineered rules, these approaches learn representations and decision functions from data. Their relevance to electron microscopy therefore extends beyond individual tasks such as denoising or segmentation. Learning-based methods provide a framework for addressing increasingly heterogeneous datasets, complex microstructures, multimodal information, and analytical relationships that are difficult to encode explicitly using conventional algorithms.

**Figure 4:**
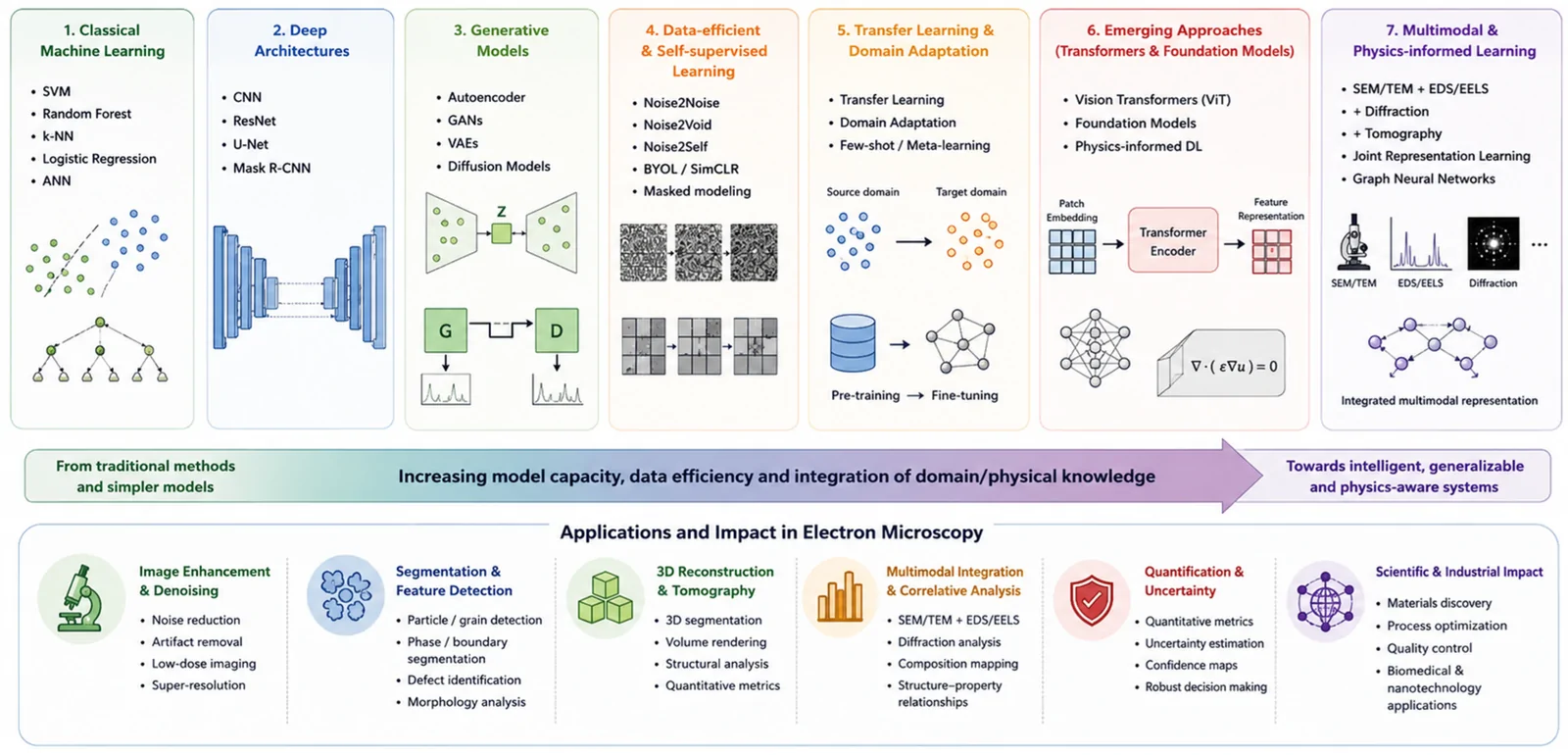
Taxonomy and methodological evolution of machine learning (ML) and deep learning (DL) approaches for electron microscopy. The framework progresses from classical machine-learning methods and deep convolutional architectures to generative models, data-efficient and self-supervised learning, transfer learning and domain adaptation, transformers and foundation models, and multimodal and physics-informed learning. The directional arrow represents the general trend toward increasing model capacity, data efficiency, and integration of domain and physical knowledge. Representative applications include image enhancement and denoising, segmentation and feature detection, three-dimensional reconstruction and tomography, multimodal interpretation, quantitative analysis, and uncertainty estimation, ultimately supporting more robust, generalizable, and interpretable electron microscopy workflows. Figure developed by the authors using Microsoft PowerPoint. No generative AI tools were used in its preparation.

The application of ML to electron microscopy has evolved from conventional classifiers based on manually engineered features toward increasingly sophisticated learning frameworks capable of extracting hierarchical representations directly from imaging data. This evolution encompasses not only deeper neural architectures but also alternative learning paradigms designed to address limited annotations, domain shifts, multimodal information, and the incorporation of physical knowledge. Figure 4 summarizes this methodological progression, organizing the principal ML and DL approaches discussed in this review and linking them to representative applications in electron microscopy and materials characterization.

As illustrated in Figure 4, this progression should not be interpreted as a strict chronological replacement of earlier methods by more recent architectures. Rather, it reflects a general trend toward increasing representational capacity, data efficiency, transferability, and integration of domain-specific and physical knowledge.

Classical ML methods remain valuable for well-defined problems and limited datasets, whereas convolutional and generative architectures enable increasingly complex image-restoration and segmentation tasks. More recent developments—including self-supervised learning, domain adaptation, transformers, foundation models, multimodal learning, and physics-informed approaches—seek to address some of the principal limitations associated with data availability, generalization across instruments and materials, and the scientific reliability of model predictions. Accordingly, the following subsections examine this methodological progression from classical ML approaches and neural-network-based image analysis to deep architectures, generative and self-supervised learning, transfer and domain-adaptation strategies, and emerging multimodal and physics-informed frameworks. This progression is presented to clarify how each methodological development addresses specific limitations of previous approaches and contributes complementary capabilities toward more robust, transferable, and scientifically reliable AI-assisted electron microscopy.

### 4.1. Classical Machine Learning Approaches

Early applications of ML in electron microscopy primarily relied on supervised learning algorithms using hand-crafted features extracted from microscopy images. Unlike modern deep learning approaches, these methods require the prior extraction of quantitative descriptors before classification or segmentation can be performed. Feature extraction therefore plays a central role and typically involves descriptors related to texture, intensity distributions, morphology, shape, or frequency-domain information [5,9].

The classical machine learning algorithms most commonly employed in electron microscopy can be grouped into several methodological families according to their learning strategy. Margin-based classifiers, represented by Support Vector Machines (SVMs), have demonstrated robust performance for mineral classification and microstructural recognition because of their ability to construct optimal decision boundaries in high-dimensional feature spaces [33,34]. Tree-based learning methods, particularly Decision Trees (DTs) and Random Forests (RFs), have been extensively used for pixel-level classification in SEM-EDS datasets due to their robustness, interpretability, and ability to handle heterogeneous feature sets [34,35]. Instance-based learning methods, such as k-Nearest Neighbors (k-NN), provide a computationally simple alternative by assigning class labels according to similarity in the feature space [9,36]. Linear probabilistic models, including Logistic Regression (LR), have frequently served as baseline classifiers because of their computational efficiency and ease of interpretation [9,37]. Finally, shallow Artificial Neural Networks (ANNs) represented one of the earliest nonlinear learning approaches applied to microscopy data, demonstrating promising performance in particle recognition and automated microstructural characterization prior to the emergence of deep learning architectures [10,29,38].

Several studies have demonstrated the effectiveness of these approaches in specific electron microscopy applications. For example, texture descriptors combined with classical supervised classifiers have been successfully employed to recognize steel microstructures in SEM images, by procedures of classification cropping into various sub-images and then applying image processing features, achieving classification accuracies of approximately 85% [39].

Similarly, Random Forest classifiers have demonstrated strong performance for pixel-level mineral classification using SEM-EDS images, reporting high F1-scores while maintaining competitive generalization capabilities compared with other classical machine learning algorithms [34]. These studies illustrate that carefully designed feature engineering strategies can produce reliable quantitative characterization under well-controlled experimental conditions. Despite these successes, classical machine learning approaches present several inherent limitations. Their predictive performance depends heavily on manually engineered features, whose effectiveness is often highly specific to a given material system or imaging condition. Consequently, transferring models between different microscopes, materials, or acquisition parameters frequently requires redesigning feature extraction pipelines and extensive parameter optimization. In addition, feature engineering and classifier training are typically performed as independent stages, limiting the ability of these methods to learn complex hierarchical image representations. These limitations reduce scalability and have ultimately motivated the transition toward deep learning frameworks capable of jointly learning feature representations and predictive models directly from raw microscopy images [5,9,13].

Table 2 groups together classical supervised machine learning algorithms that are commonly useful for microscopy image analysis. Prior to the widespread adoption of deep learning, these methods relied on manually engineered feature descriptors extracted from microscopy images for classification, segmentation, and quantitative characterization tasks. While they demonstrated satisfactory performance in specific applications, their dependence on feature engineering limited their generalization across different materials systems and imaging conditions.

### 4.2. Deep Learning for Image Enhancement

Deep learning methods, particularly convolutional neural networks, have demonstrated strong performance in image enhancement tasks, including denoising, deblurring, and super-resolution. In electron microscopy, these capabilities are valuable for improving image quality under low-dose and rapid-acquisition conditions, where SNR is often limited. Supervised CNN-based denoising approaches have been applied to suppress noise in SEM/TEM images while preserving fine structural details. When paired datasets are available (noisy inputs and high-quality references), DL models generally outperform classical filtering methods in both objective metrics (e.g., PSNR, SSIM) and qualitative assessments [40,41]. Similarly, DL-based super-resolution methods can reconstruct higher-resolution images from low-resolution inputs, potentially reducing electron dose by avoiding repeated or prolonged acquisition.

However, the requirement for paired training data is a major obstacle in practical microscopy settings, where acquiring corresponding low- and high-quality images for the same field of view may be infeasible due to drift, sample instability, or beam damage. To address this constraint, unsupervised and self-supervised strategies—including autoencoders and generative adversarial neural networks—have been explored to learn latent representations of clean images or realistic degradation processes without explicit ground-truth targets [4].

### 4.3. Segmentation and Morphological Analysis

Accurate segmentation of microstructural features is critical for quantitative characterization. Deep learning-based segmentation models, such as U-Net, Mask R-CNN, and other fully convolutional architectures, have been increasingly adopted due to their ability to capture spatial context and delineate complex structures. Deep learning has been applied to particle and feature segmentation in SEM images, including cases with irregular morphologies and non-uniform contrast. ML-driven auto-segmentation has been demonstrated in application-specific contexts, such as asphaltene deposition studies [29]. For nanoparticle characterization, DL-based pipelines can support automated measurement of particle size and morphology and can remain effective even under challenging imaging conditions involving overlap and heterogeneous contrast [10,13].

Nevertheless, segmentation remains limited by the availability of high-quality annotations, which are costly to generate. This has motivated research into weakly supervised, semi-supervised, and transfer learning approaches, as well as workflows that combine partial labels with model uncertainty or active learning to reduce annotation burden [11].

### 4.4. Learning-Based Analysis and Intelligent Electron Microscopy Workflows

Beyond conventional morphology analysis, learning-based methods have increasingly been applied to multimodal microscopy datasets, including SEM–EDS, TEM-based spectroscopic imaging, electron energy-loss spectroscopy (EELS), and energy-dispersive X-ray spectroscopy (EDS). The integration of structural and compositional information enables more comprehensive materials characterization while simultaneously increasing the complexity and dimensionality of the acquired data. Consequently, machine learning algorithms have become valuable tools for extracting meaningful information from these heterogeneous datasets [5,34]. For example, pixel-level mineral classification in SEM–EDS images has been successfully demonstrated using both classical machine learning and deep learning models, enabling automated compositional mapping with high classification accuracy despite challenges associated with noise, heterogeneous phase distributions, and class imbalance [34]. These approaches significantly reduce the manual effort required for compositional analysis while improving reproducibility across large microscopy datasets.

An important consideration in the combined analysis of SEM images and EDS elemental maps is the difference in their effective information depths and spatial resolutions. Secondary electron images predominantly provide surface-sensitive morphological information, whereas characteristic X-rays used for EDS analysis may originate from a substantially larger interaction volume beneath the specimen surface, depending on accelerating voltage, material composition, density, and specimen geometry [21]. Consequently, morphological boundaries observed in SEM images may not coincide precisely with compositional boundaries identified through EDS mapping. This mismatch can introduce uncertainty into pixel-level classification, phase identification, and multimodal segmentation, particularly for small particles, thin layers, and heterogeneous microstructures. Therefore, machine-learning approaches combining SEM and EDS information should account for differences in signal-generation volumes and spatial resolution when interpreting classification results and validating quantitative measurements.

Recent advances in deep learning have also enabled significant improvements in image reconstruction and resolution enhancement. De Haan et al. proposed a generative adversarial neural network (GAN)-based framework capable of transforming low-resolution SEM images into images exhibiting spatial frequency characteristics comparable to high-resolution acquisitions [42]. Their results demonstrated that deep learning-based super-resolution can recover unresolved structural details while simultaneously reducing acquisition time, electron charging effects, and beam-induced specimen damage, thereby improving both imaging throughput and experimental efficiency. These findings highlight the potential of learning-based image reconstruction not only to improve image quality but also to reduce the operational constraints associated with high-resolution SEM imaging. Beyond image reconstruction, recent developments have expanded the role of artificial intelligence toward the automation of complete SEM workflows. Clusiau et al. [12] developed an AI-assisted framework that integrates automated image acquisition, feature tracking, beam repositioning, and quantitative characterization into a unified workflow for scanning electron microscopy. Their approach enables automatic relocation of regions of interest during sequential acquisitions, reducing manual intervention while improving acquisition efficiency and experimental throughput. Such workflow-oriented automation demonstrates that machine learning can support not only image interpretation but also microscope operation itself, contributing to reduced operational costs, improved reproducibility, and more efficient utilization of high-value microscopy facilities [12].

In three-dimensional characterization, deep learning methods have also been investigated for denoising, reconstruction, and artifact suppression in EDS and EELS tomography. Numerical reconstruction techniques for low-dose EDS tomography have demonstrated the severe impact of Poisson noise and incomplete sampling on elemental reconstruction accuracy, emphasizing the need for dose-efficient computational imaging strategies [6]. More recently, learning-based reconstruction methods have shown considerable promise for improving throughput, reconstruction fidelity, and dose efficiency in three-dimensional elemental mapping, particularly when combined with advanced detector technologies, multimodal imaging, and physics-informed reconstruction frameworks [3,4].

Three-dimensional (3D) electron microscopy extends quantitative materials characterization beyond two-dimensional projections and surface-based measurements by providing access to spatially resolved microstructural information. However, the underlying acquisition and reconstruction principles differ substantially among serial sectioning, focused ion beam–scanning electron microscopy (FIB-SEM), transmission electron microscopy (TEM) tomography, and electron holography. In serial-section FIB-SEM, successive material layers are removed and imaged to generate an ordered stack of cross-sectional images, from which three-dimensional morphology can be reconstructed through alignment, segmentation, and volumetric assembly. This approach is particularly valuable for characterizing interconnected pores, grain boundaries, phase distributions, and heterogeneous microstructures. TEM tomography, by contrast, reconstructs three-dimensional structural information from projections acquired at different specimen tilt angles, introducing challenges associated with limited angular coverage, noise, and alignment accuracy. Electron holography provides complementary quantitative information by retrieving electron-wave phase shifts associated with electrostatic and magnetic fields. In a representative experimental study, Tanigaki et al. [43] demonstrated the three-dimensional reconstruction of electrostatic potential distributions in platinum nanoparticles using off-axis electron holographic tomography, achieving a reported spatial resolution of 1.5 nm. More recently, Tanigaki [44] reviewed advances in quantitative and three-dimensional electron holography, including the reconstruction of electrostatic potentials and magnetic-field distributions. These studies demonstrate the increasing capabilities of advanced electron microscopy for volumetric and field-sensitive characterization, while also highlighting the need to distinguish the physical information provided by different reconstruction modalities.

The increasing dimensionality and complexity of three-dimensional electron microscopy datasets create opportunities for machine learning (ML) and deep learning (DL) at different stages of the computational workflow. Learning-based approaches can support image denoising, inter-slice registration, segmentation, reconstruction from incomplete measurements, and the quantitative extraction of three-dimensional microstructural descriptors. Nevertheless, the appropriate computational strategy depends on the physical origin of the acquired signal and the specific reconstruction objective. In FIB-SEM, learning-based segmentation has been investigated for volumetric materials characterization. For example, Fend et al. [45] demonstrated the reconstruction of highly porous structures from FIB-SEM images using a deep neural network trained on synthetic image data, thereby reducing dependence on manually annotated experimental datasets. Similarly, Hirabayashi et al. [46] investigated deep-learning-based semantic segmentation of FIB-SEM datasets acquired from complex polycrystalline ceramic materials. Their study evaluated fully convolutional networks and U-Net architectures, reporting an intersection-over-union value of up to 94.6% for U-Net under the investigated conditions. The resulting segmentation supported the reconstruction of large-scale three-dimensional microstructures, illustrating the potential of learning-based methods to facilitate quantitative characterization of heterogeneous materials. However, these findings should not be interpreted as evidence that deep learning universally improves volumetric reconstruction, since model performance remains dependent on the imaging conditions, training-data representativeness, annotation accuracy, and characteristics of the material system.

A particularly important limitation of FIB-SEM tomography arises when imaging porous or partially transparent structures. Because secondary electrons may originate from surfaces located behind the nominal milling plane, the recorded image can contain contributions from subsurface features visible through pores. These so-called shine-through or pore-back effects complicate the distinction between solid material, exposed pore surfaces, and structures located deeper within the specimen. Consequently, conventional intensity thresholding may produce incorrect phase assignments, distort pore connectivity, or introduce artificial structures along the sectioning direction. Salzer et al. [47] quantitatively compared segmentation strategies using simulated FIB-SEM images of porous media and demonstrated that shine-through artifacts can lead to substantial errors in the spatial localization of reconstructed structures. Furthermore, Fager et al. [48] developed an optimized FIB-SEM tomography workflow for soft, porous, and poorly conducting materials, addressing charging, shadowing, cross-sectioning artifacts, and overlapping grayscale intensities. Their approach incorporated random-forest-based segmentation to distinguish solid and pore regions in reconstructed polymer microstructures. These studies demonstrate that reliable volumetric characterization requires both appropriate acquisition conditions and segmentation strategies capable of accounting for modality-specific artifacts. Although ML-based approaches may improve phase discrimination under challenging imaging conditions, their effectiveness depends on whether the training data adequately represent the relevant imaging artifacts, specimen geometries, and material characteristics.

Taken together, these examples emphasize the importance of distinguishing computationally enhanced reconstruction from experimentally validated three-dimensional information. AI-assisted segmentation, denoising, and restoration may reduce manual processing effort, improve consistency across large image stacks, and facilitate the quantitative analysis of complex microstructures. However, these approaches cannot automatically compensate for missing projections, irreversible milling damage, beam-induced structural changes, or systematic acquisition artifacts. Furthermore, models trained on simulated or narrowly defined datasets may exhibit reduced reliability when applied to different pore morphologies, material compositions, microscope configurations, or acquisition conditions. Similar concerns apply to electron tomography and holographic reconstruction, where incomplete angular sampling, phase-retrieval uncertainties, and noise may introduce ambiguities that cannot be resolved through algorithmic complexity alone. Reliable three-dimensional characterization therefore requires physically informed reconstruction strategies, careful uncertainty assessment, and validation against independent measurements or representative reference structures whenever possible. The integration of conventional reconstruction algorithms, learning-based processing, and modality-specific physical constraints represents a promising direction for increasingly automated electron microscopy, provided that computationally generated features are not interpreted as experimentally established structures without sufficient supporting evidence.

Despite these advances, learning-based analysis of spectroscopic and tomographic datasets continues to face several important challenges. Publicly available multimodal microscopy datasets remain scarce, limiting reproducible benchmarking and comparative evaluation of competing algorithms. Moreover, the reliability and generalization capability of learning-based models strongly depend on the quality, consistency, and representativeness of the microscopy data used during training. Variations in imaging parameters, noise levels, magnification, electron dose, preprocessing procedures, and other acquisition conditions can introduce distributional differences between training and experimental datasets, potentially degrading model performance when applied to previously unseen data. Indeed, recent studies using experimentally acquired HRTEM datasets have demonstrated that neural- network performance can be sensitive to variations in microscope parameters and data preprocessing, emphasizing the importance of carefully curated and representative training datasets [49]. Therefore, appropriate image acquisition, preprocessing, quality control, and dataset curation are essential for developing robust models capable of reliable deployment across different experimental conditions. Furthermore, three-dimensional reconstruction requires substantial computational resources, while reliable validation often depends on physically meaningful ground-truth data that are difficult or impossible to obtain experimentally. Consequently, future research should address not only the development of standardized datasets, self-supervised learning strategies, and physics-informed deep learning models, but also the establishment of rigorous data-quality and curation criteria capable of improving model generalization while maintaining scientific interpretability [4,5].

**Figure 5:**
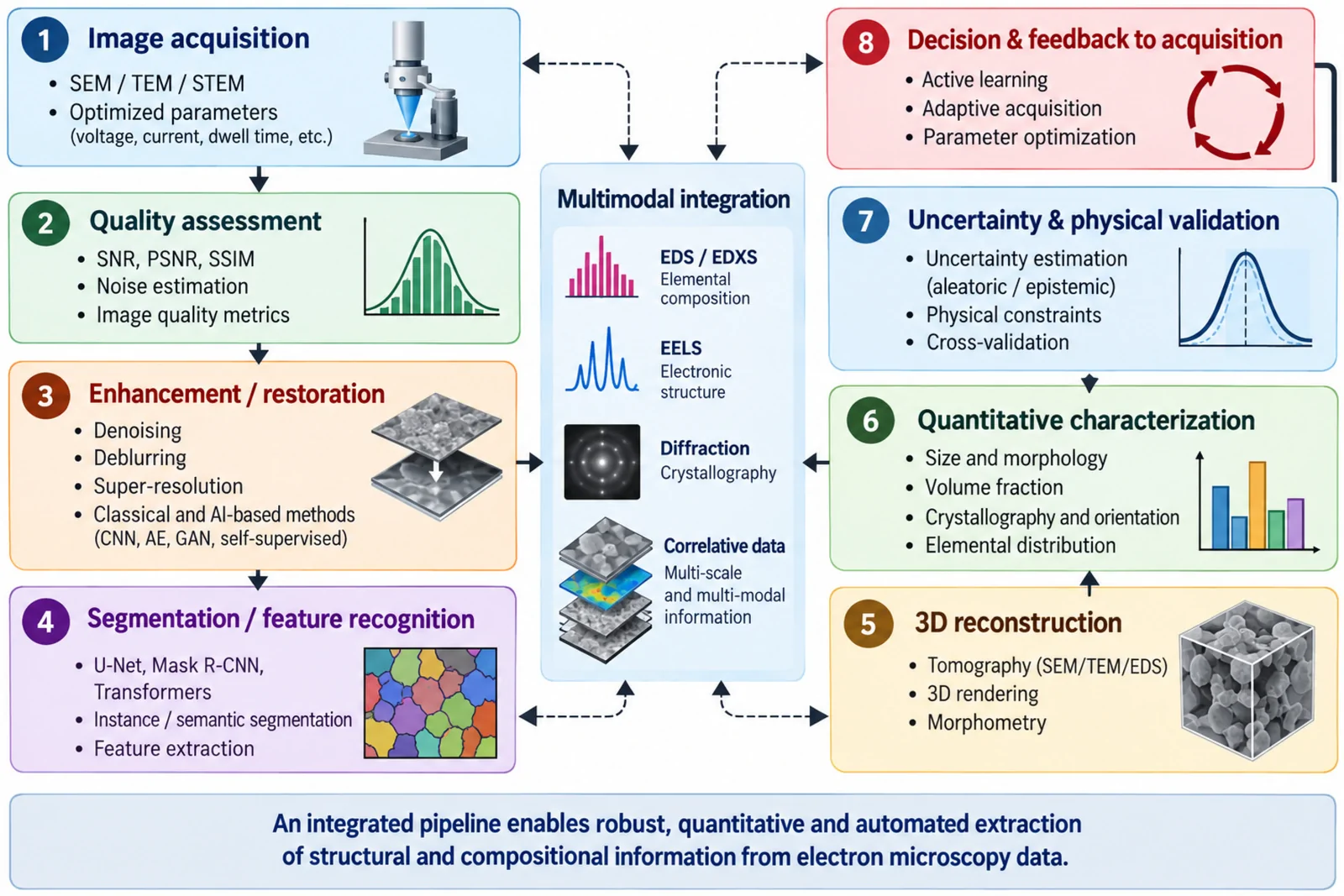
Conceptual workflow for intelligent and multimodal electron microscopy, connecting image acquisition, quality assessment, enhancement and restoration, segmentation and feature recognition, three-dimensional reconstruction, quantitative characterization, uncertainty estimation and physical validation, and feedback-assisted acquisition. Multimodal integration of EDS/EDXS, EELS, diffraction, and correlative data illustrates how complementary structural and compositional information can be incorporated throughout the analytical pipeline to support robust, quantitative, and increasingly automated materials characterization. The figure was developed by the authors using Google Gemini as a conceptual illustration. The microscopy-like images, graphical elements, and analytical outputs are schematic representations and do not constitute original experimental data or validated quantitative results. Figure created by the authors using Google Gemini.

The increasing integration of ML into electron microscopy is progressively extending its role beyond isolated image-processing or classification tasks. Enhancement, segmentation, multimodal analysis, three-dimensional reconstruction, and quantitative characterization can instead be considered interconnected components of a broader computational workflow. In this context, information generated at one stage can support subsequent analytical steps, while uncertainty estimates and physically meaningful constraints can provide additional criteria for assessing the reliability of automated outputs. Figure 5 summarizes this integrated perspective, illustrating how acquisition, image processing, multimodal information, quantitative analysis, and validation can be combined within an intelligent electron microscopy workflow.

Importantly, the workflow represented in Figure 5 is not intended as a strictly sequential pipeline. Multimodal information from EDS, EELS, diffraction, and correlative measurements can be incorporated at different stages to complement morphological information derived from SEM, TEM, or STEM images. Similarly, uncertainty estimation and physical validation can be used to identify unreliable predictions, whereas quantitative outputs may ultimately provide feedback for adaptive acquisition or parameter optimization. This closed-loop perspective represents an important step toward more autonomous microscopy workflows in which acquisition and computational analysis become increasingly interconnected.

In summary, implementing such integrated workflows requires computational architectures capable of learning complex image representations, preserving spatial information, and adapting to diverse analytical tasks. The following section therefore examines the principal deep learning architectures that provide the computational foundation for many of these capabilities.

### 4.5. Deep Learning Architectures for Electron Microscopy

The success of deep learning in electron microscopy is largely attributable to the development of neural network architectures capable of learning hierarchical feature representations directly from image data. Unlike traditional machine learning approaches, which generally depend on manually engineered descriptors and separate feature-extraction stages, deep learning models can jointly learn relevant image features and task-specific representations during training. This capability has enabled their application to increasingly complex electron microscopy tasks, including automated feature detection, segmentation, image restoration, quantitative microstructural analysis, and high-throughput characterization [5,9,49,50].

Convolutional neural networks (CNNs) remain among the most widely employed deep learning architectures for electron microscopy image analysis. Through convolutional operations, CNNs learn local spatial features such as edges, textures, particle boundaries, defects, and crystallographic contrast, while successive layers progressively construct higher-level representations of complex microstructural patterns. CNN-based and related deep neural network architectures have demonstrated strong performance in applications ranging from particle and morphology recognition to defect identification, segmentation, and image enhancement in SEM and TEM datasets [10,13,50,51]. Recent developments have further extended these architectures through the integration of synthetic training data, self-supervised learning, domain-aware strategies, and confidence estimation, addressing important limitations associated with scarce annotations and model generalization across different materials and experimental conditions [49,50,51].

For image segmentation applications, encoder–decoder architectures such as U-Net have become particularly relevant because of their ability to perform pixel-level classification while preserving spatial information. Originally developed for biomedical image segmentation, U-Net combines a contracting path for multi-scale feature extraction with an expanding path and skip connections that recover spatial details required for accurate localization [52]. In electron microscopy, U-Net and related architectures have been employed to identify and delineate microstructural features such as individual particles, grains and grain boundaries, pores, phases, inclusions, and defects. These segmentation results can subsequently support quantitative characterization tasks, including particle counting and size-distribution analysis, grain-size measurement, porosity estimation, phase-fraction determination, defect localization, and morphological assessment of micro- and nanostructures.

For instance, deep learning-based segmentation has been applied to particle identification in SEM images across different magnifications and particle length scales, facilitating automated quantitative particle characterization [53]. U-Net-based semantic segmentation has also been investigated using three-dimensional electron microscopy datasets of polycrystalline ceramic materials, enabling the identification and quantitative analysis of grains, porosity, and secondary phases in geometrically complex microstructures [46].

**Figure 6:**
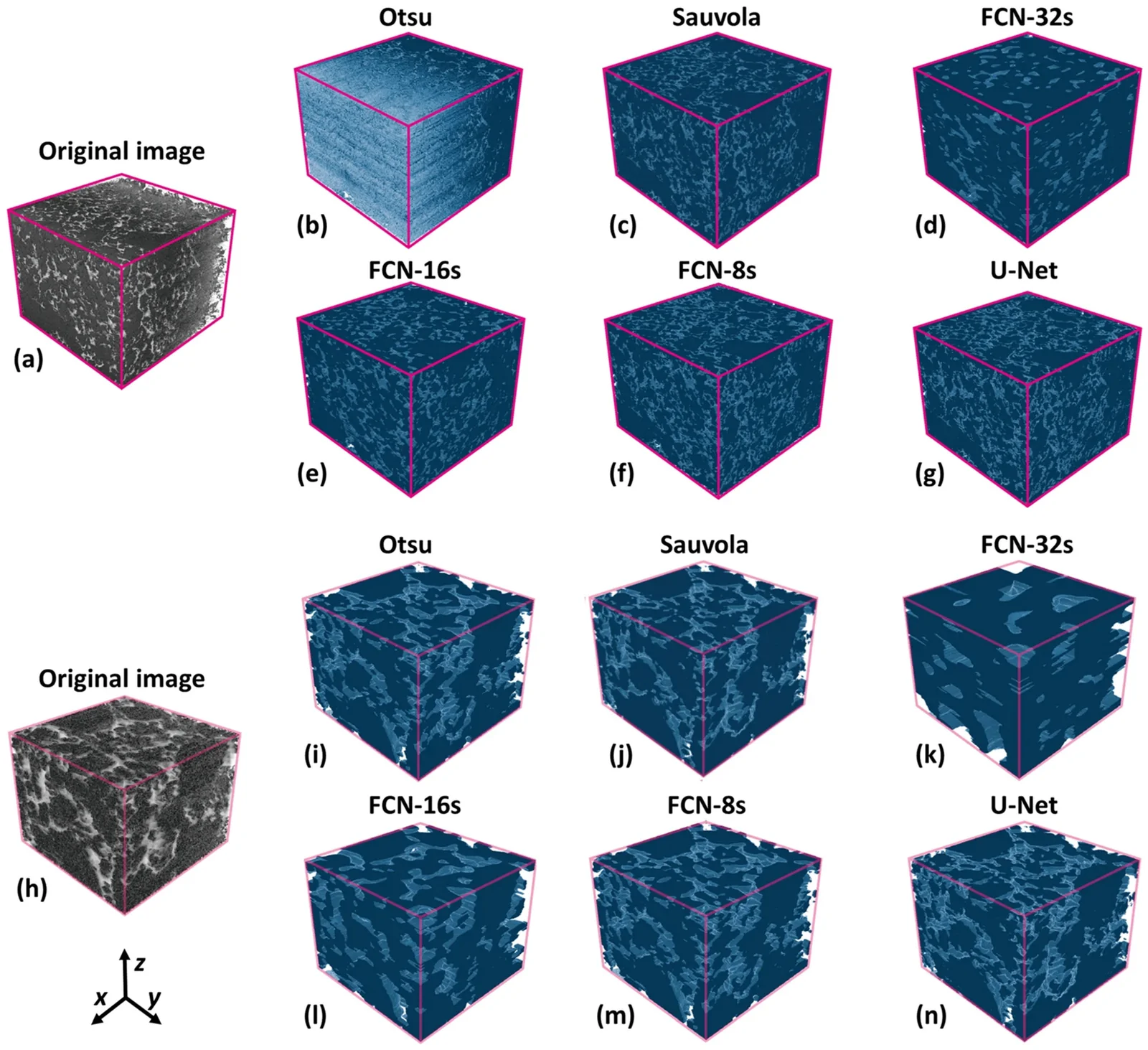
Three-dimensional reconstructions obtained from conventional thresholding methods (Otsu and Sauvola) and deep-learning-based segmentation models (FCN-32s, FCN-16s, FCN-8s, and U-Net). Panels (**a**–**g**) show a reconstructed volume of 768 × 768 × 620 voxels, while panels (**h**–**n**) show an enlarged central region of 256 × 256 × 206 voxels. The comparison illustrates differences in the reconstructed microstructures and highlights the relevance of conventional segmentation methods as computationally simpler alternatives. Reproduced from [46], subject to the original publication’s reuse terms. Copyright 2024, NPJ.

Hirabayashi et al. [46] systematically compared conventional thresholding methods (Otsu and Sauvola) with deep-learning-based segmentation architectures (FCN-32s, FCN-16s, FCN-8s, and U-Net) using FIB-SEM images of a polycrystalline ceramic material. Their analysis included three-dimensional reconstruction from 620 serially acquired secondary electron images. As illustrated in Figure 6, Sauvola produced visually continuous three-dimensional reconstructions comparable to those obtained using certain neural-network architectures. However, the quantitative evaluation revealed important differences. Although Sauvola achieved the highest recall, it also produced false-positive classifications that resulted in an overestimation of the superconducting phase fraction. In contrast, U-Net achieved the highest overall segmentation accuracy, with an Intersection over Union (IoU) of 94.6%, while U-Net and FCN-8s provided phase-fraction estimates close to those obtained from expert-annotated reference images.

These findings demonstrate that conventional adaptive thresholding can remain competitive for specific microstructural segmentation tasks, particularly when computational simplicity and the absence of model-training requirements are important considerations. Nevertheless, the advantages of deep-learning-based approaches become more evident when accurate identification of fine defects, suppression of imaging artifacts, and quantitative phase characterization are required. The original study reported approximately two hours of neural-network training and only a few minutes for automated segmentation of the 620-image dataset, although it did not establish a direct, standardized runtime comparison with Sauvola. Therefore, the selection of a segmentation method should be based on quantitative accuracy, microstructural complexity, robustness to imaging artifacts, and computational requirements rather than visual reconstruction quality alone [46].

More recently, U-Net architectures combined with EDS-guided labeling have been used for phase segmentation in SEM images of solid oxide cell electrodes, allowing the discrimination of porosity and multiple material phases with high segmentation accuracy while substantially accelerating the analysis compared with semi-manual procedures [54]. These studies demonstrate the applicability of U-Net-based segmentation to materials systems in which conventional thresholding and edge-based methods become unreliable because of low contrast, irregular boundaries, overlapping or agglomerated particles, imaging artifacts, and heterogeneous microstructures.

More advanced architectures, particularly residual neural networks (ResNets), were developed to address the degradation problem that may arise when increasingly deep neural networks are trained. In this context, degradation does not refer to deterioration of the input image, but rather to a decline in network optimization and training performance as additional layers are introduced. In conventional deep networks, increasing depth does not necessarily lead to improved accuracy; beyond a certain point, deeper architectures may exhibit higher training error than their shallower counterparts, even in the absence of overfitting. He et al. demonstrated this phenomenon experimentally and introduced residual learning as a strategy to facilitate the optimization of substantially deeper networks [55]. Instead of requiring each group of layers to directly learn a complete underlying mapping, ResNets learn a residual mapping relative to the input through identity-based skip connections. These connections provide alternative pathways for information and gradient propagation across the network, facilitating optimization and mitigating difficulties associated with training very deep architectures [55]. In electron microscopy, these characteristics are particularly relevant for tasks requiring the extraction of complex hierarchical features, including microstructural classification, defect recognition, segmentation, denoising, and image restoration. Residual architectures can therefore support the analysis of complex microscopy datasets by enabling deeper feature extraction while maintaining effective network optimization [5].

Object detection frameworks such as Mask R-CNN further extend segmentation capabilities by simultaneously identifying individual objects and generating pixel-level masks. These methods are particularly attractive for nanoparticle characterization, where overlapping structures and irregular morphologies complicate traditional segmentation procedures. By combining localization and segmentation within a unified framework, Mask R-CNN can provide quantitative measurements of particle size, shape, and distribution with minimal user intervention [10,13].

More recently, transformer-based architectures have emerged as a promising alternative to conventional CNNs. Unlike convolutional models that primarily capture local spatial relationships, transformers employ self-attention mechanisms capable of modeling long-range dependencies across entire images. This capability may be advantageous for analyzing complex hierarchical microstructures, multimodal datasets, and large-scale microscopy images. Although still relatively unexplored in electron microscopy, transformer-based approaches represent an important direction for future research [4].

### 4.6. Generative Models and Image Reconstruction

Generative deep learning models have attracted increasing attention for image reconstruction, restoration, and enhancement in microscopy. Unlike discriminative models, which primarily learn mappings for tasks such as classification or segmentation, generative approaches model characteristics of the underlying data distribution and can therefore be used to reconstruct, synthesize, denoise, or enhance image information. Recent developments have expanded this field beyond conventional generative adversarial networks (GANs) toward more generalizable generative and foundation-model-based restoration frameworks, particularly for microscopy applications where noise, limited resolution, and acquisition constraints remain major challenges [9,56].

Autoencoders represent one of the established neural architectures for image reconstruction and denoising. These networks encode input images into a lower-dimensional latent representation from which the image is subsequently reconstructed, allowing relevant structural information to be separated from noise and other unwanted variations. Denoising autoencoders and related deep neural network approaches are particularly attractive for low-dose electron microscopy, where reducing electron exposure can protect beam-sensitive specimens but simultaneously decreases the signal-to-noise ratio. Recent studies continue to demonstrate the effectiveness of deep learning-based denoising for electron microscopy, including approaches capable of improving TEM image quality while preserving structural information required for quantitative interpretation [8,40,41].

Generative adversarial networks (GANs) have further expanded the capabilities of learning-based image reconstruction [57]. GANs employ adversarial training between a generator, which produces reconstructed or synthetic images, and a discriminator, which attempts to distinguish generated outputs from real observations. This framework has been applied to denoising, deblurring, contrast enhancement, and super-resolution microscopy [58], where recovering high-frequency structural information from degraded or low-resolution observations is particularly important [9]. More broadly, recent generative approaches to microscopy restoration increasingly combine adversarial, multi-scale, and conditional generative strategies to improve reconstruction quality and generalization across different imaging conditions [56]. Nevertheless, the application of generative models to scientific microscopy requires rigorous validation, since visually plausible reconstructions do not necessarily guarantee faithful recovery of the underlying physical structure. This consideration is particularly critical in electron microscopy, where artificially generated features could lead to incorrect interpretation of defects, interfaces, atomic arrangements, or other microstructural characteristics [40].

Learning-based reconstruction, including GAN-based super-resolution, is particularly relevant to dose- and time-constrained electron microscopy. Since excessive electron exposure may damage sensitive materials, reducing acquisition dose or acquisition time is often desirable. Deep learning-based reconstruction can partially compensate for the resulting loss of image quality or spatial resolution, providing a promising route toward more dose-efficient microscopy workflows [4,42].

Generative approaches also show potential for creating synthetic training datasets that can alleviate the scarcity of annotated microscopy images. Synthetic image generation may improve model robustness, support data augmentation strategies, and facilitate transfer learning in applications where acquiring large experimental datasets is difficult or prohibitively expensive [5].

### 4.7. Self-Supervised and Unsupervised Learning

A major limitation in microscopy-oriented deep learning is the scarcity of annotated training datasets. Generating pixel-level labels for segmentation or obtaining paired degraded-reference images is frequently impractical due to experimental constraints such as beam damage, sample drift, and acquisition time limitations. Consequently, self-supervised and unsupervised learning strategies have emerged as attractive alternatives [4,5].

Self-supervised and reference-free learning strategies exploit statistical relationships within the available data to construct training objectives without requiring conventional clean ground-truth images. Among the pioneering approaches, Noise2Noise demonstrated that image restoration networks can be trained using pairs of independently corrupted observations of the same underlying signal, eliminating the requirement for clean target images under appropriate statistical assumptions [59]. Noise2Void extended this concept by introducing a blind-spot training strategy that enables denoising directly from individual noisy images, without requiring either clean targets or paired noisy observations [60]. Similarly, Noise2Self introduced a general self-supervised framework based on statistical independence of noise across measurement dimensions, allowing denoising performance to be learned and evaluated using only noisy observations [61]. These approaches are particularly relevant to microscopy, where acquiring paired clean and degraded images under identical experimental conditions can be difficult or impossible. By exploiting the statistical properties of noisy observations, such methods reduce dependence on experimentally acquired ground-truth data and provide promising alternatives for denoising low-dose and noise-limited microscopy datasets [4].

Unsupervised learning approaches similarly seek to discover latent structure within microscopy datasets. Clustering algorithms, autoencoders, and representation-learning frameworks can identify meaningful patterns and group similar microstructural features without explicit labels. Such methods are particularly relevant for exploratory materials research, where previously unseen structures may be encountered and prior annotation may not exist [9].

The ability of unsupervised learning methods to exploit large quantities of unlabeled microscopy data is particularly important given the increasing adoption of automated acquisition systems and high-throughput microscopy platforms. As the volume of microscopy data continues to expand, unsupervised learning is expected to play a central role in future materials characterization workflows [5].

Furthermore, self-supervised learning aligns closely with the practical realities of electron microscopy, where generating extensive ground-truth datasets remains one of the most significant barriers to the widespread deployment of deep learning systems. Consequently, these approaches represent one of the most promising research directions for next-generation intelligent microscopy platforms [4].

### 4.8. Transfer Learning and Domain Adaptation

Transfer learning has emerged as an effective strategy for addressing one of the most significant challenges in electron microscopy image analysis: the limited availability of large annotated datasets. Unlike natural image domains, where millions of labeled images are available for training deep neural networks, electron microscopy datasets are often small, highly specialized, and expensive to generate. Consequently, training deep learning models from scratch may lead to overfitting and poor generalization performance [5].

Transfer learning has emerged as an effective strategy for addressing the limited availability of large, accurately annotated datasets in electron microscopy. Instead of training deep neural networks entirely from scratch, models previously trained on large-scale image collections can be adapted to microscopy-specific tasks by reusing learned feature representations and subsequently fine-tuning selected network layers. Early convolutional layers generally encode relatively transferable visual patterns, such as edges, contours, textures, and geometric structures, whereas deeper layers capture increasingly task-specific representations. By exploiting these previously learned features, transfer learning can reduce the amount of microscopy-specific training data required and accelerate model convergence, making it particularly valuable for SEM and TEM applications in which the generation and expert annotation of sufficiently large datasets are costly and time-consuming. Recent studies have demonstrated the effectiveness of pretrained and transfer-learning strategies for automated analysis of electron microscopy images, including microstructural classification, feature recognition, and segmentation, while also highlighting the importance of adapting pretrained representations to differences between natural-image and microscopy domains [62,63,64].

Several studies have demonstrated the effectiveness of transfer learning and pre-trained deep neural networks for classification, segmentation, and quantitative feature-recognition tasks in microscopy. Stuckner et al. demonstrated that encoders pre-trained on a large and diverse microscopy dataset can improve microstructure segmentation compared with conventional ImageNet-based initialization, highlighting the advantages of domain-specific pre-training for materials characterization [62]. Transfer-learning strategies have also been applied directly to TEM datasets. Lu et al. combined self-supervised pre-training with transfer learning to perform classification and segmentation of nanowire morphologies using only a limited number of labeled TEM images, demonstrating the potential of previously learned representations to reduce annotation requirements [63]. More recently, Jo et al. developed a transfer-learning-based semantic segmentation framework specifically for noisy and limited wafer TEM images, in which pre-training followed by process-specific fine-tuning was used to address data scarcity and improve boundary identification for automated semiconductor measurements [64]. Collectively, these studies demonstrate that transfer learning is particularly valuable in materials science and electron microscopy, where datasets are frequently restricted to specific materials, imaging modalities, acquisition parameters, and experimental conditions, making the generation of large and comprehensively annotated datasets both time-consuming and costly. Transfer learning therefore provides a practical strategy for leveraging previously acquired representations while reducing microscopy-specific data requirements and facilitating the development of robust models for specialized characterization tasks.

Despite its advantages, transfer learning does not completely eliminate domain-specific challenges. Electron microscopy images differ substantially from general-purpose photographic images, such as those contained in large-scale datasets like ImageNet, in terms of contrast formation, noise statistics, texture distributions, spatial characteristics, and the underlying physical mechanisms of image formation. Consequently, feature representations learned from general-purpose image datasets may not always transfer optimally to SEM and TEM applications, particularly when higher-level features are strongly associated with the source domain. This domain discrepancy has motivated increasing interest in domain adaptation and microscopy-specific pre-training strategies designed to reduce distributional differences between source and target datasets and improve model generalization [5,49,62].

Domain adaptation encompasses a variety of approaches that seek to improve model generalization across different imaging conditions, microscopes, laboratories, or materials systems. These techniques may involve adversarial learning, feature alignment, or self-supervised representation learning to reduce sensitivity to domain-specific variations. In electron microscopy, domain adaptation is particularly relevant because image characteristics can vary significantly according to experimental setup, such as accelerating voltage, detector configuration, sample preparation procedures, and acquisition parameters.

The importance of domain adaptation becomes even more evident in high-throughput characterization workflows. As microscopy facilities increasingly generate data from diverse instrument setups and materials classes, models capable of maintaining performance across heterogeneous datasets will become essential. Future research is therefore expected to focus on developing transferable representations that can generalize across imaging modalities while preserving sensitivity to relevant microstructural information [4].

Overall, transfer learning and domain adaptation provide a practical pathway for overcoming two major barriers to the broader implementation of AI in electron microscopy: the limited availability of labeled microscopy data and the loss of model performance when acquisition conditions, instruments, or material systems change. By enabling knowledge learned in one domain to be adapted to another, these approaches can reduce retraining requirements while improving model transferability, reproducibility, and scalability across research laboratories and industrial characterization environments. Their principal contribution therefore extends beyond improving predictive performance: they provide a methodological foundation for developing AI-assisted microscopy systems that can operate more reliably across heterogeneous experimental conditions.

### 4.9. Physics-Informed Learning and Emerging Trends

Although conventional deep learning approaches have achieved remarkable success in image enhancement, segmentation, and classification, most existing models remain fundamentally data-driven. These methods typically learn statistical relationships from training datasets without explicitly incorporating knowledge of the underlying physical processes governing image formation. While effective under many circumstances, purely data-driven approaches may generate physically inconsistent predictions, particularly when training data are limited or when imaging conditions differ from those encountered during training [5].

To address these limitations, increasing attention has been directed toward physics-informed machine learning frameworks. These approaches seek to integrate physical knowledge, mathematical constraints, or imaging models directly into the learning process. By embedding information regarding electron–matter interactions, detector characteristics, noise statistics, or reconstruction physics, physics-informed models can improve robustness, interpretability, and generalization performance [4,65].

One promising direction involves the incorporation of known image degradation mechanisms into neural network training. In electron microscopy, image quality is strongly influenced by factors such as shot noise, beam broadening, drift, charging effects, and detector limitations. Physics-informed models can exploit prior knowledge of these processes to constrain neural network outputs and reduce the likelihood of generating unrealistic image features. Such constraints are particularly valuable in low-dose imaging applications, where preserving scientific validity is often more important than maximizing visual appearance [6].

Another emerging area is the integration of machine learning with tomographic reconstruction. Three-dimensional characterization techniques frequently involve incomplete, noisy, or sparsely sampled datasets, creating challenging inverse problems. Traditional reconstruction algorithms often require substantial computational resources and may produce artifacts under low-dose conditions. Advanced computational reconstruction methods have demonstrated the potential to accelerate reconstruction while improving image quality and dose efficiency under challenging acquisition conditions [3,14].

Transformer architectures and foundation models represent a rapidly growing research direction for automated image analysis, representation learning, and segmentation in electron microscopy. In contrast to conventional convolutional architectures, which primarily capture local spatial patterns through convolutional operations, vision transformers employ self-attention mechanisms to model relationships between spatially distant image regions, enabling the representation of both local and global contextual information [66]. This capability is particularly relevant to electron microscopy, where structural information may extend across multiple spatial scales and complex microstructural regions. Recent studies have demonstrated the potential of transformer-based and foundation-model approaches for electron microscopy segmentation. Abebe et al. adapted the Segment Anything Model (SAM) for zero-shot segmentation of atomic-scale transmission electron micrographs, demonstrating the potential of general-purpose vision foundation models for microstructural analysis while also highlighting the challenges associated with domain-specific image characteristics [67]. Similarly, Shi et al. developed ShapeMamba-EM, a foundation-model fine-tuning approach for three-dimensional electron microscopy segmentation that incorporates local morphological descriptors to address the domain gap between general-purpose pretrained models and EM datasets [68]. More recently, Xing et al. introduced RETINA, a pretrained TransUNet-based framework that combines convolutional feature extraction with transformer-based global-context modeling and demonstrated improved segmentation performance across multiple electron microscopy datasets [69]. Collectively, these studies indicate that transformer-based and foundation-model approaches provide promising strategies for developing more transferable and adaptable electron microscopy analysis frameworks. Their extension toward the integration of complementary SEM, TEM, EDS, EELS, diffraction, and other characterization data represents a promising future direction for multimodal materials characterization.

The growing adoption of multimodal learning is particularly relevant for next-generation materials characterization. Future microscopy platforms are expected to generate increasingly diverse datasets containing complementary structural, chemical, crystallographic, and spectroscopic information. Machine learning frameworks capable of integrating these heterogeneous data sources may enable more comprehensive characterization of complex materials by jointly exploiting information that would otherwise be analyzed independently. Such multimodal approaches could also facilitate the identification of previously unrecognized correlations between microstructure, composition, processing conditions, and material properties, ultimately contributing to a deeper understanding of structure–property relationships [1,34].

Finally, explainable artificial intelligence (XAI) has become an important area of research in both medical imaging and materials characterization. As machine learning models are increasingly used to support scientific decision-making, understanding the rationale behind model predictions becomes essential. Techniques such as Grad-CAM and related saliency- or attention-based visualizations can help identify image regions contributing to classification decisions, thereby improving interpretability [70].

Overall, physics-informed learning, multimodal integration, transformer architectures, and explainable artificial intelligence collectively represent some of the most promising directions for future research in electron microscopy. These developments have the potential to move machine learning beyond simple image enhancement toward comprehensive, physically grounded, and scientifically interpretable characterization frameworks.

In summary, ML and DL have expanded SEM and TEM image analysis capabilities by enabling increasingly automated, objective, and scalable characterization workflows. Deep learning methods are particularly effective at capturing complex nonlinear relationships in microscopy data, supporting image enhancement, segmentation, feature recognition, and quantitative analysis beyond the capabilities of many conventional image-processing approaches. However, most current models remain specialized to specific datasets, acquisition conditions, microscope configurations, or material classes, and their performance may deteriorate when applied to data that differ from those used during training [49,62]. A major objective for future research is therefore the development of instrument- and material-agnostic models capable of maintaining reliable performance across different microscope platforms, imaging parameters, laboratories, and classes of materials. Achieving this level of generalization will require representative and diverse training datasets, data-efficient learning strategies, domain adaptation, transfer learning, and standardized evaluation protocols capable of assessing model robustness across experimental domains [49,62]. These advances will be essential for transitioning ML-based methods from application-specific solutions toward transferable and reliable computational tools that can be integrated into intelligent routine electron microscopy workflows independently of specific instruments or material systems.

Taken together, the evidence reviewed in this section suggests that the next major advance in AI-assisted electron microscopy will depend less on increasing model complexity alone than on improving transferability, physical consistency, uncertainty awareness, and validation beyond the original training domain. Similar challenges have already emerged in medical and biological imaging, where AI models must remain reliable despite variations in imaging equipment, acquisition protocols, institutions, and the characteristics of the analyzed biological or patient populations. The strategies developed in these fields to improve data representativeness, model generalization, uncertainty assessment, and external validation can therefore provide methodological guidance for addressing comparable sources of variability in electron microscopy, including differences among microscopes, acquisition conditions, laboratories, and material systems. These cross-disciplinary parallels are examined in Section 5.

## 5. Machine Learning in Medical and Biological Imaging: Transferable Insights for Electron Microscopy

Medical and biological imaging provide a valuable comparative framework for understanding the opportunities and limitations of artificial intelligence in electron microscopy. Although biomedical imaging and materials characterization differ fundamentally in their physical mechanisms, spatial scales, specimens, and analytical objectives, both fields face related computational challenges. Imaging data may be affected by noise, acquisition-dependent variability, complex contrast mechanisms, heterogeneous structures, limited annotations, and instrument-specific characteristics. Moreover, the increasing dimensionality and volume of imaging data have created a common need for automated methods capable of extracting reproducible and quantitatively meaningful information.

ML and DL have been extensively investigated in radiology, ultrasound, biological imaging, and spectroscopy for tasks including detection, classification, segmentation, reconstruction, image enhancement, and quantitative analysis [71,72]. The relevance of these developments to electron microscopy lies not in the direct transfer of models between fundamentally different imaging modalities, but in the methodological strategies developed to address recurring computational problems. Biomedical AI has confronted challenges involving limited or biased datasets, generalization across equipment and institutions, operator-dependent acquisition, three-dimensional (3D) reconstruction, multimodal integration, uncertainty, and the need for rigorous external validation [71,73,74]. Closely related challenges occur in SEM and TEM when computational models are transferred across microscopes, acquisition parameters, laboratories, material systems, or characterization modalities.

Accordingly, the following subsections examine selected biomedical applications as cross-disciplinary case studies. Radiological imaging illustrates the importance of dataset quality, domain generalization, and external validation; ultrasound provides relevant examples of operator dependence, complex image formation, physics-guided learning, and 3D reconstruction; and spectroscopy demonstrates how ML can extract meaningful information from high-dimensional molecular and chemical datasets. These examples are subsequently integrated with the learning paradigms and architectures discussed in Section 4 to identify methodological principles that may be adapted to the specific physics and quantitative requirements of electron microscopy.

### 5.1. Machine Learning in Radiological Imaging: Methodological Insights into Generalization and Validation

Radiological imaging modalities such as computed tomography (CT), magnetic resonance imaging (MRI), and digital radiography are among the most extensively studied domains for ML-based image analysis. These techniques generate large and complex datasets that must be interpreted by clinicians, making them particularly suitable for automated pattern recognition, segmentation, and predictive modeling. ML and DL algorithms have consequently been applied to disease classification, lesion detection, anatomical and pathological structure segmentation, and prognosis prediction [71,75,76].

Convolutional neural networks (CNNs) have played a particularly important role because of their ability to learn hierarchical representations directly from imaging data. Rather than relying exclusively on manually engineered descriptors, CNN-based models can identify complex spatial patterns associated with anatomical structures or pathological alterations [71]. The increasing availability of large imaging datasets and computational resources has further accelerated their application across radiological modalities.

The rapid development of AI for chest radiography and CT during the COVID-19 pandemic provides an instructive example of both the potential and limitations of data-driven medical imaging. Although numerous studies reported high classification performance for identifying abnormalities associated with SARS-CoV-2 infection [75,76,77], systematic evaluations identified important methodological weaknesses, including dataset selection bias, confounding variables, insufficient external validation, and limited reproducibility [73]. These findings demonstrated that strong performance within a development dataset does not necessarily translate into reliable predictions across institutions or patient populations. More broadly, successful clinical translation requires representative datasets, transparent evaluation procedures, and validation under conditions that differ from those used during model development [73,78]. Differences in scanner manufacturers, acquisition protocols, reconstruction procedures, and institutional practices may introduce domain shifts that compromise model generalization.

Comparable generalization challenges arise in electron microscopy, where SEM and TEM images vary according to microscope and detector configuration, accelerating voltage, beam current, electron dose, magnification, specimen preparation, acquisition protocols, and subsequent image processing [5,49]. Variations in material composition, morphology, crystallography, and electron–matter interactions further influence image contrast and feature representation. Consequently, models trained on a restricted combination of instruments, acquisition conditions, and material classes may learn dataset-specific correlations rather than physically transferable microstructural characteristics [49,62]. This limitation is particularly relevant to automated characterization across laboratories and industrial environments, where changes in detector geometry, noise characteristics, or material morphology may reduce segmentation and classification reliability. Accordingly, predictive performance within a single dataset should not be interpreted as evidence of generalizability across experimental conditions.

Strategies developed in biomedical imaging to address these limitations provide useful guidance for electron microscopy. Multi-institutional datasets have been used to increase diversity, while transfer learning, domain adaptation, and external validation have been employed to evaluate and improve model robustness. Comparable strategies in electron microscopy could include multi-instrument and multi-laboratory datasets, explicit evaluation across acquisition conditions, and validation on material classes excluded from model development. Importantly, external validation should not be treated merely as a final reporting step but as part of the experimental design used to determine whether a model has learned transferable physical characteristics.

Radiological imaging has also demonstrated the value of combining imaging information with complementary clinical variables. ML models integrating imaging biomarkers with patient metadata and longitudinal information can provide more comprehensive representations of disease processes than models based on individual data sources [74]. Although the nature of the information differs, an analogous opportunity exists in materials characterization, where SEM and TEM images can be integrated with spectroscopy, diffraction, processing parameters, experimental metadata, and material-property measurements.

The principal contribution of radiological AI to electron microscopy is therefore methodological rather than evidence of direct model transfer. Experience in radiology highlights the importance of representative training data, independent validation, and robustness across acquisition environments. In materials characterization, these principles should be evaluated through multi-instrument, multi-laboratory, and cross-material testing, with particular attention to the preservation of physically meaningful microstructural information. Such validation is essential for progressing from dataset-specific demonstrations toward reproducible AI-assisted electron microscopy workflows.

### 5.2. Machine Learning for Ultrasound Image Analysis

Ultrasound represents another important biomedical modality in which ML has demonstrated substantial potential. Unlike CT and MRI, ultrasound image acquisition is highly dependent on operator experience, probe positioning, acquisition parameters, and patient-specific conditions. Images are also frequently affected by speckle noise, heterogeneous contrast, artifacts, and poorly defined anatomical boundaries, which can complicate both visual interpretation and automated analysis [72].

ML and DL methods have been investigated for segmentation of anatomical structures, detection and classification of abnormalities, tissue characterization, image-quality enhancement, and computer-assisted diagnosis [72]. CNN-based architectures are particularly effective for extracting relevant features from noisy images and can support real-time applications in which computational predictions provide guidance during image acquisition or interpretation. This interaction between acquisition and analysis is particularly relevant to the development of increasingly automated imaging systems.

The operator dependence of ultrasound provides a useful conceptual parallel with electron microscopy. SEM and TEM acquisition similarly requires decisions regarding field-of-view selection, focus, magnification, beam parameters, detector configuration, dwell time, and acquisition geometry. Experienced microscopists continuously adapt these parameters according to specimen characteristics and the information required from the experiment. Consequently, both fields face the challenge of transforming expert-dependent acquisition procedures into more reproducible computational workflows.

Hybrid approaches combining signal processing with ML have also been investigated in ultrasound to improve reconstruction and noise suppression. In this modality, speckle is associated with coherent wave interference and therefore cannot be treated simply as additive noise. Similarly, electron microscopy images are influenced by modality-specific physical processes, including electron counting statistics, detector response, electron–matter interactions, charging, and contrast formation. Although these mechanisms differ fundamentally from ultrasound image formation, both fields illustrate the importance of incorporating acquisition physics into computational enhancement. Consequently, denoising and restoration methods developed for one modality cannot be assumed to preserve physically meaningful information when applied directly to another without appropriate adaptation and validation.

A particularly relevant development is the reconstruction of 3D anatomical information from sequences of two-dimensional (2D) ultrasound images. Freehand 3D ultrasound can generate volumetric representations from sequential B-scans, but accurate reconstruction requires reliable estimation of the spatial position and orientation of individual frames. Probe motion, speckle decorrelation, out-of-plane displacement, and accumulated tracking errors can introduce inconsistencies between consecutive images and compromise the reconstructed volume [79,80].

DL approaches have therefore been developed to estimate inter-frame motion and spatial relationships directly from ultrasound sequences, enabling trackerless reconstruction and reducing dependence on external tracking devices [79,80]. More recently, physics-guided DL approaches have incorporated prior knowledge of ultrasound image formation and speckle characteristics into sensorless 3D reconstruction [81]. These developments illustrate an important evolution from models that learn exclusively from data toward hybrid approaches in which physical knowledge constrains or informs the learning process.

Three-dimensional ultrasound reconstruction and electron microscopy tomography share computational challenges related to image registration, incomplete measurements, volumetric reconstruction, and uncertainty propagation. However, their acquisition principles are fundamentally different. Freehand ultrasound reconstruction involves the spatial arrangement of successive acoustic B-scans, whereas TEM tomography reconstructs specimen structure from electron projections acquired at different tilt angles. Serial-section FIB-SEM follows another acquisition strategy, in which successive material layers are removed and imaged to construct a volumetric dataset. Consequently, although learning-based approaches developed for ultrasound may provide methodological insights into registration, reconstruction, and uncertainty estimation, their direct applicability to electron microscopy must be established through modality-specific evaluation. In electron microscopy, reconstruction errors can affect the apparent geometry of particles, pores, interfaces, phases, and defects, thereby influencing subsequent quantitative measurements.

The architectures discussed in Section 4 can contribute at multiple stages of this process. CNNs and encoder–decoder architectures can support denoising and segmentation, while generative and physics-informed models can contribute to reconstruction under incomplete or noisy measurements. Learning-based registration can assist alignment, and uncertainty estimation can identify regions in which the reconstructed volume is less reliable. Rather than treating these operations independently, their integration could enable connected computational pipelines in which information generated during one stage informs subsequent reconstruction or analysis.

Three-dimensional reconstruction also illustrates why numerical or visual improvement alone is insufficient for scientific imaging. A reconstructed volume may appear structurally plausible while containing features that are unsupported by the experimental measurements. Physics-guided learning is therefore particularly relevant because it offers a mechanism for incorporating acquisition geometry, image-formation constraints, or known physical relationships into data-driven reconstruction.

Ultrasound imaging therefore offers two relevant methodological insights for electron microscopy: reducing operator dependence through acquisition-aware computational assistance and improving reconstruction reliability by incorporating physical constraints into learning-based methods. These principles may inform the development of integrated electron microscopy workflows involving denoising, registration, volumetric reconstruction, segmentation, and uncertainty assessment. Nevertheless, their implementation requires adaptation to electron–matter interactions, acquisition geometry, specimen characteristics, and the quantitative objectives of materials characterization, rather than direct transfer of ultrasound-specific reconstruction models.

### 5.3. Machine Learning-Enhanced Spectroscopy for Biomedical and Materials Analysis

Beyond conventional imaging modalities, ML has become increasingly important for the analysis of spectroscopic data in medical and biological research. Spectroscopy provides molecular and chemical information that is complementary to morphological imaging but generates high-dimensional datasets in which relevant information may be distributed across multiple wavelengths, frequencies, or spectral features. ML provides strategies for dimensionality reduction, feature extraction, classification, and identification of relationships that may be difficult to recognize using conventional univariate analysis.

Classical approaches such as principal component analysis (PCA), support vector machines (SVMs), k-nearest neighbors (k-NN), and random forests (RFs), together with neural-network-based methods, have been applied to complex spectroscopic datasets [82,83]. These methods can identify subtle combinations of spectral features rather than relying exclusively on individual peaks or manually selected spectral regions.

Raman spectroscopy represents a well-established example in biomedical and biological analysis. Raman spectra contain molecular information associated with proteins, lipids, nucleic acids, and other biochemical constituents, providing a biochemical fingerprint of the analyzed specimen [82,83]. When combined with ML, these spectral patterns can be used to distinguish physiological and pathological states and to identify molecular changes that may precede visible morphological alterations.

ML-assisted Raman spectroscopy has consequently been investigated across a variety of biomedical applications, including cancer detection and classification, tumor grading, infectious diseases, monitoring of therapeutic response, and cellular characterization [82,84,85,86,87,88,89,90]. Studies of oral and gastric cancer, for example, demonstrate how complex spectral signatures can be transformed into classification features, while investigations at the cellular level illustrate the potential for label-free identification of molecular phenotypes [84,85,86,87,88,89]. Raman-based analysis of biological tissues and extracellular matrices further demonstrates the capability of computational spectroscopy to identify compositional differences that may not be readily distinguishable through morphology alone [90].

The relevance of biomedical Raman spectroscopy to electron microscopy lies primarily in shared computational challenges rather than equivalent spectroscopic mechanisms or directly transferable diagnostic models. Raman spectroscopy probes vibrational information through inelastic light scattering, whereas energy-dispersive X-ray spectroscopy (EDS) detects characteristic X-rays generated by electron–matter interactions, and electron energy-loss spectroscopy (EELS) measures the energy lost by transmitted electrons. Consequently, these techniques differ in their signal formation, spectral interpretation, spatial resolution, and measurement limitations. Nevertheless, their datasets can present related computational challenges, including overlapping spectral features, low signal-to-noise ratios, correlated variables, instrument-dependent measurements, and sensitivity to preprocessing [82,83]. Approaches involving dimensionality reduction, feature extraction, classification, and multimodal analysis therefore offer methodological insights for electron microscopy, provided that they are adapted to the relevant physical processes and validated against appropriate experimental measurements.

In electron microscopy spectroscopy, ML-based approaches can support dimensionality reduction, spectral classification, denoising, and the identification of relationships between compositional information and microstructural features. For example, ML analysis of low-loss EELS data has been investigated for extracting physically meaningful information from spatially resolved spectroscopic measurements [91]. More broadly, clustering and classification methods may assist the identification of compositionally distinct regions, while learning-based denoising and representation-learning strategies offer opportunities for analyzing low-count or high-dimensional spectral datasets. However, the applicability of these methods depends on the acquisition conditions, signal statistics, spectral calibration, and availability of suitable reference measurements. Improvements in computational classification or visual quality should therefore not be interpreted automatically as improvements in quantitative compositional accuracy.

A further methodological parallel concerns the integration of morphological and spectroscopic information. In biomedical applications, molecular signatures can complement anatomical or cellular imaging; similarly, electron microscopy can combine SEM/TEM morphology with EDS, EELS, diffraction, and experimental metadata to obtain complementary descriptions of material structure and composition. Joint analysis may support the characterization of particles, phases, defects, and interfaces using information beyond image appearance alone. However, reliable multimodal interpretation requires attention to spatial registration, differences in sampling volumes and signal-generation mechanisms, and the uncertainty associated with each measurement. The principal transferable insight is therefore the use of ML to integrate complementary, high-dimensional information while preserving the physical meaning and limitations of the individual characterization techniques.

### 5.4. Transferable Methodological Insights for Electron Microscopy and Materials Characterization

The biomedical examples discussed above reveal that the principal value of cross-disciplinary comparison does not lie in transferring individual algorithms directly between fundamentally different imaging modalities. Rather, it lies in identifying methodological principles that have already become critical in another data-intensive imaging field and determining how those principles can be adapted to the specific physical and analytical requirements of electron microscopy.

A first insight concerns data availability and data efficiency. Biomedical imaging has demonstrated the importance of large, representative datasets while simultaneously confronting the practical difficulty of obtaining extensive expert annotations. Electron microscopy faces an analogous constraint because manual annotation of particles, grains, phases, pores, defects, interfaces, and other microstructural features requires specialized knowledge and can be prohibitively time-consuming. The data-efficient approaches reviewed in Section 4—including transfer learning, self-supervised learning, data augmentation, and domain-specific pre-training—provide complementary strategies for exploiting limited labeled data and the considerably larger volumes of unlabeled SEM and TEM data routinely generated in microscopy laboratories [60,61,62].

The experience of biomedical imaging also indicates that increasing dataset size alone does not guarantee generalization. Diversity in acquisition conditions, instruments, specimen types, and target populations must be represented adequately during model development and validation. Applied to electron microscopy, this principle suggests that future datasets should capture variation across microscopes, detectors, accelerating voltages, magnifications, specimen-preparation procedures, laboratories, and material classes. Dataset diversity should therefore be considered an experimental-design variable rather than merely a requirement for increasing the number of training images.

A second finding concerns architecture selection according to the analytical problem rather than model novelty. The architectures reviewed in Section 4 provide complementary capabilities. CNNs and residual architectures can support feature extraction, classification, defect recognition, and restoration; encoder–decoder architectures such as U-Net are particularly appropriate for pixel-level segmentation; autoencoders and generative approaches can contribute to denoising and reconstruction; self-supervised approaches can exploit unlabeled data; and transformers and foundation models provide mechanisms for modeling longer-range relationships and transferring representations across datasets and tasks. These approaches should therefore not be regarded as competing stages in a linear technological progression, but as components that can be selected and combined according to the experimental problem.

This distinction is important because many electron microscopy workflows involve several interconnected tasks. Image restoration may precede segmentation, segmentation may enable quantitative measurements, spectroscopic information may support classification, and uncertainty estimates may determine whether an automated result should be accepted or reviewed by an expert. The future role of AI may therefore depend less on identifying a single universally superior architecture and more on integrating complementary learning paradigms within end-to-end or modular characterization workflows.

Three-dimensional reconstruction illustrates the distinction between methodological parallels and experimentally demonstrated applications. Studies of ultrasound reconstruction have shown how learning-based approaches can assist spatial registration and volumetric reconstruction, including through physics-guided strategies [79,80,81]. Electron tomography and serial-section FIB-SEM involve different acquisition geometries and image-formation mechanisms, but share computational challenges related to alignment, incomplete or noisy measurements, artifact suppression, and quantitative validation. As discussed in Section 4, learning-based reconstruction methods have also been investigated in electron microscopy, including approaches incorporating physical constraints [65]. These examples support the relevance of physics-aware computational strategies, although their implementation and performance must be validated separately for each microscopy modality and materials system.

A third consideration is generalization across acquisition environments and material systems. As demonstrated in biomedical imaging, strong internal model performance does not guarantee reliability under changes in equipment, protocols, or data distributions [73]. In electron microscopy, transfer learning, domain adaptation, self-supervision, and domain-specific pre-training may improve robustness [62], but their effectiveness must be assessed using independent datasets acquired across different microscopes, laboratories, imaging conditions, and material classes. Instrument- and material-independent performance should therefore be treated as an experimental validation objective rather than an assumed property of increasingly complex neural networks.

A fourth critical consideration is the importance of multimodal integration. Biomedical AI increasingly combines images with spectral, longitudinal, molecular, and clinical information. Materials characterization offers a comparable opportunity through the integration of SEM/TEM images with EDS, EELS, diffraction, processing parameters, and experimental metadata. Rather than analyzing these signals independently, multimodal learning may identify relationships among morphology, composition, crystallography, processing history, and material properties that are not evident from any individual modality [82,83].

A methodological insight concerns interpretability, uncertainty, and trustworthy AI. In biomedical applications, computational predictions may influence clinical decisions; consequently, high predictive performance alone is insufficient when the confidence and reliability of a prediction cannot be assessed. Although the consequences and regulatory environments differ, scientific interpretation in materials characterization similarly requires confidence that computational outputs represent experimentally supported information.

Uncertainty-aware DL has already been investigated for SEM particle segmentation and 3D electron microscopy segmentation, enabling the identification of predictions whose reliability decreases under blur, agglomeration, morphological variability, noise, or ambiguous boundaries [53,92]. Such approaches could become particularly important in automated and high-throughput microscopy. Rather than forcing an algorithm to provide a definitive result for every input, uncertainty estimates could identify conditions in which additional acquisition, alternative processing, or expert intervention is required.

This capability introduces an important connection between uncertainty and autonomous microscopy. A future intelligent characterization system could use uncertainty not only to describe confidence after analysis but also as feedback for experimental decision-making. Regions associated with high uncertainty could trigger additional imaging, modified acquisition parameters, higher-resolution measurements, complementary spectroscopy, or human review. In this way, AI could evolve from passive post-processing toward active support of the characterization process.

The integration of these principles with the methods discussed in Section 4 provides a framework for evaluating the methodological relevance of biomedical AI to electron microscopy. Table 3 summarizes representative applications in biomedical imaging and electron microscopy, together with their supporting references. Importantly, similarities in computational strategies do not necessarily demonstrate direct transfer of trained models between imaging modalities. While biomedical applications illustrate transferable methodological principles and validation challenges, studies such as the deep-learning-based segmentation of complex ceramic materials using FIB-SEM reported by Hirabayashi et al. [46] provide direct evidence of AI applications within electron microscopy. Accordingly, the examples in Table 3 should be interpreted within their respective experimental contexts, distinguishing demonstrated applications from methodological parallels that require further modality-specific validation.

**Figure 7:**
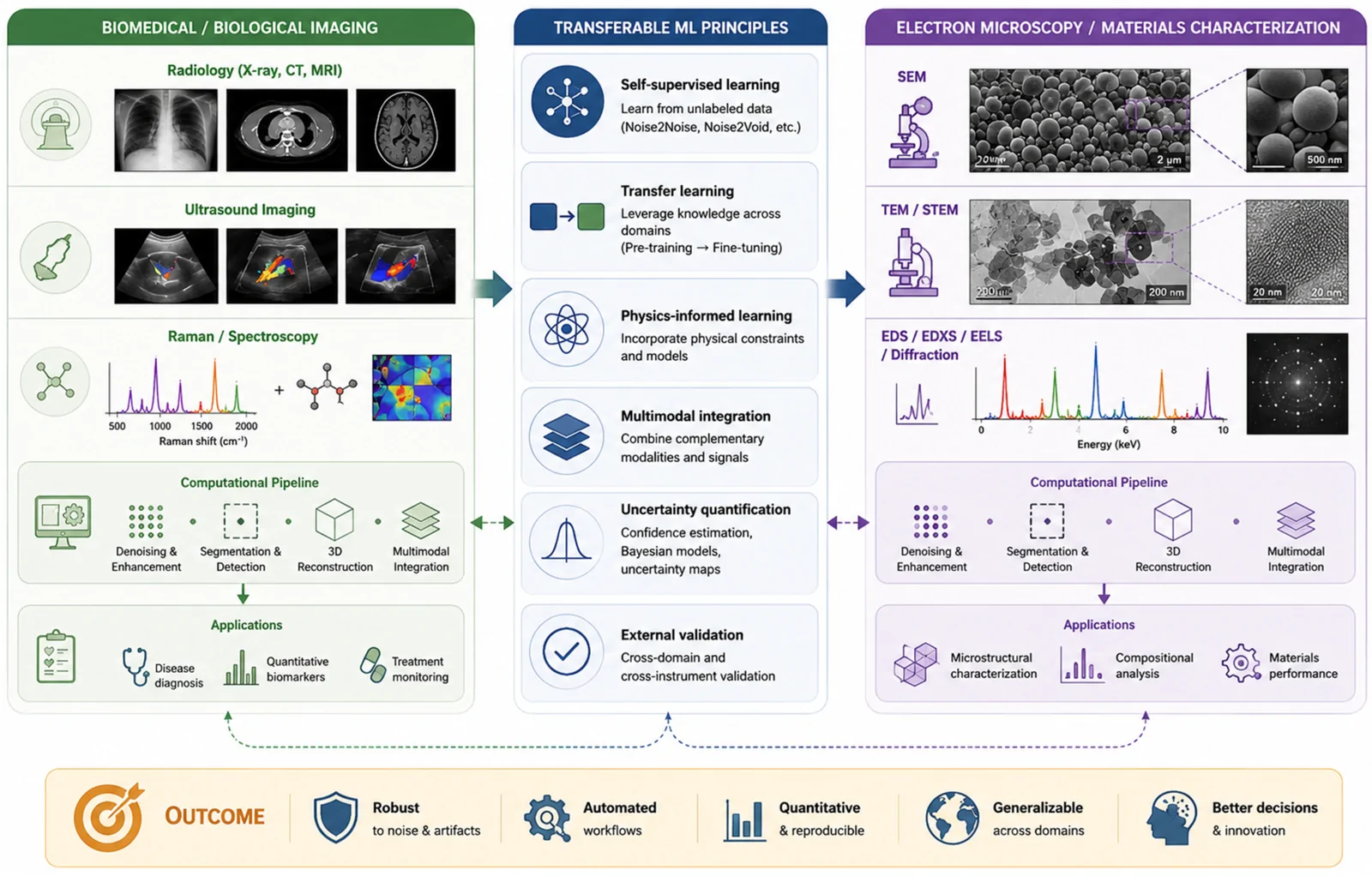
Conceptual framework illustrating methodological connections between biomedical imaging and electron microscopy for materials characterization. Radiological imaging, ultrasound, and Raman spectroscopy provide examples of computational strategies that address challenges also encountered in electron microscopy, including limited annotated data, acquisition-dependent variability, image degradation, multimodal information, and model reliability. The central panel summarizes six relevant methodological principles: self-supervised learning, transfer learning, physics-informed learning, multimodal integration, uncertainty quantification, and external validation. The framework illustrates potential methodological adaptation rather than demonstrated direct transfer of trained models or equivalent performance across imaging modalities. Application to SEM, TEM/STEM, EDS/EDXS, EELS, and diffraction-based characterization requires modality-specific adaptation and experimental validation. The figure was developed by the authors using Google Gemini. All microscopy-like images, medical imaging examples, spectra, and graphical elements are illustrative representations and do not constitute original experimental data or validated quantitative results.

Figure 7 summarizes this cross-disciplinary framework and the principal methodological strategies that may be adapted to electron microscopy.

As illustrated in Figure 7, the relationship between biomedical imaging and electron microscopy is primarily methodological. Self-supervised learning, transfer learning, physics-informed approaches, multimodal integration, uncertainty quantification, and external validation offer complementary strategies for addressing data limitations, reconstruction challenges, and model reliability. However, their applicability to materials characterization must be established through adaptation to electron–matter interactions, microscopy-specific acquisition conditions, and the physical quantities being measured. The framework therefore identifies promising directions for methodological development rather than demonstrating direct transferability of individual biomedical models.

Overall, the medical and biological applications examined in this section provide a complementary perspective on the future role of AI in electron microscopy. Their principal contribution is not evidence that identical models can be used across disciplines, but demonstration that reliable AI requires coordinated attention to data quality, domain generalization, physical constraints, multimodal information, uncertainty, and external validation. Radiology demonstrates why strong internal performance must be complemented by independent validation; ultrasound illustrates how operator dependence, image-formation physics, and 3D reconstruction can be incorporated into learning-based workflows; and biomedical spectroscopy demonstrates how high-dimensional and complementary signals can be integrated to reveal information unavailable from individual modalities.

When these insights are combined with the architectures and learning paradigms reviewed in Section 4, a broader direction for electron microscopy emerges: the transition from isolated models optimized for individual image-processing tasks toward integrated, physics-aware, uncertainty-aware, and multimodal characterization systems. Such systems should ultimately be evaluated not only by their ability to enhance or segment images, but by their capacity to preserve physically meaningful information, generalize across instruments and material classes, integrate complementary measurements, quantify uncertainty, and support reproducible scientific decisions. This cross-disciplinary synthesis represents a central contribution of the present review and provides a methodological framework for identifying which advances from biomedical AI can be adapted to accelerate robust and increasingly autonomous electron microscopy.

Achieving this transition, however, still requires overcoming important limitations related to data availability, generalization, computational requirements, physical reliability, standardization, and integration with microscopy instrumentation. These challenges and their implications for future development are discussed in the following section.

## 6. Limitations, Open Challenges, and Future Directions

Despite the rapid development of ML and DL for electron microscopy, the transition from successful proof-of-concept demonstrations to reliable, transferable, and routinely deployable characterization tools remains incomplete. The evidence reviewed in the preceding sections suggests that future progress will depend not simply on increasing model complexity or computational capacity, but on addressing a set of interconnected challenges involving data quality and representativeness, generalization across experimental domains, physical consistency, uncertainty, multimodal integration, computational scalability, and standardized validation. These challenges become increasingly important as AI moves from isolated post-processing tasks toward integrated workflows capable of supporting quantitative and potentially autonomous materials characterization.

Data availability remains a fundamental constraint, although the challenge should be understood in terms of data quality, diversity, and traceability rather than dataset size alone. Many supervised learning approaches require extensive annotated datasets, whereas manual labeling of SEM and TEM images is time-consuming and requires specialized knowledge. Obtaining paired datasets for restoration tasks can be even more difficult because acquiring low- and high-quality images of exactly the same field of view may be compromised by specimen drift, beam-induced changes, contamination, or morphological evolution during repeated acquisition [26,27]. These constraints are particularly important under low-dose conditions, where the experimental objective is precisely to minimize electron exposure. Furthermore, large datasets acquired using a single microscope, specimen type, or acquisition protocol may contain substantial numbers of images while still representing only a narrow experimental domain. Dataset size should therefore not be interpreted as a substitute for experimental diversity.

Future dataset development should consequently emphasize representativeness and provenance. Relevant acquisition metadata—including microscope configuration, detector type, accelerating voltage, beam current, magnification, dwell time, specimen preparation, and processing history—should accompany image data whenever possible. Such information would make it possible to determine whether model performance reflects genuine microstructural learning or correlations with acquisition-specific characteristics. Multi-instrument, multi-laboratory, and multi-material datasets would also provide a stronger basis for assessing transferability. The lessons discussed in Section 5 reinforce this requirement: as demonstrated in biomedical imaging, extensive internal datasets do not necessarily guarantee reliable performance when the deployment domain differs from the development environment.

The scarcity of annotated and paired data simultaneously creates a strong motivation for data-efficient learning paradigms. Self-supervised and unsupervised learning can exploit the large volumes of unlabeled microscopy data generated during routine experiments, while weak supervision and transfer learning can reduce dependence on dense expert annotation. Synthetic data and simulation may further complement experimental datasets by providing controlled examples with known parameters or ground truth. Nevertheless, synthetic training data must reproduce the relevant physics and statistical properties of experimental microscopy sufficiently well to avoid introducing a new simulation-to-experiment domain gap. The objective should therefore not be simply to replace experimental data, but to combine experimental, simulated, and unlabeled information strategically according to the task.

Generalization across imaging conditions represents a second major challenge. Models developed using narrowly defined datasets may exhibit substantial performance degradation when applied to different microscopes, detectors, accelerating voltages, magnifications, specimen-preparation procedures, noise conditions, or material systems. This domain-shift problem is especially important for electron microscopy because image intensity and contrast are intrinsically coupled to electron–matter interactions and acquisition geometry. A model may therefore learn statistical regularities that perform well within the training distribution but are not invariant to experimental changes [13].

Transfer learning, domain adaptation, self-supervised pre-training, and foundation-model approaches provide promising strategies for reducing this dependence, but architectural sophistication alone cannot demonstrate generalization. Instrument- and material-agnostic performance should be treated as an experimental validation objective rather than an assumed property of a model. Models intended for broad use should therefore be evaluated explicitly on instruments, laboratories, acquisition conditions, and material classes that were not represented during training. This shift from internal performance toward external validation is essential if AI-assisted microscopy is to progress from dataset-specific demonstrations toward reproducible characterization tools.

A closely related challenge concerns the distinction between computational plausibility and physical fidelity. Deep-learning models, particularly highly expressive restoration and generative architectures, may produce visually convincing reconstructions containing structural features that are weakly supported or entirely unsupported by the experimental measurements, a phenomenon commonly described as AI hallucination. This risk has been documented in electron microscopy. Vincent et al. [93] demonstrated that deep-learning-based denoising of atomic-resolution TEM images could introduce spurious Pt surface atomic columns that were absent from the reference structure, highlighting the potential for false-positive atomic identification. Furthermore, Mohan et al. [40] emphasized that conventional image-quality metrics may not adequately capture scientifically meaningful structural errors in deep-learning-based electron microscopy denoising, underscoring the need for evaluation criteria that assess consistency with the experimental measurements.

In cryo-electron microscopy, Bepler et al. [19] also cautioned that denoising networks may introduce hallucinated information influenced by their training data, potentially compromising subsequent structural reconstruction. These findings demonstrate that improvements in visual quality do not necessarily guarantee accurate structural interpretation. Accordingly, AI-enhanced images should be evaluated against the original measurements, relevant image-formation physics, and, where feasible, independent experimental evidence. Particular caution is required when restored images are used for particle identification, defect analysis, segmentation, or quantitative characterization, since artificial or omitted features may propagate into downstream measurements and scientific conclusions. Collectively, these documented failure cases illustrate the current maturity of AI-assisted electron microscopy: although learning-based methods can substantially improve image interpretability and automate selected analytical tasks, their outputs cannot yet be treated as independently verified representations of the underlying microstructure. Their readiness for routine scientific characterization therefore depends on task-specific validation, representative training data, uncertainty assessment, and consistency with the original experimental measurements.

Physics-informed learning provides one route toward addressing this limitation. Incorporating knowledge of electron–matter interactions, detector response, acquisition geometry, noise statistics, or reconstruction physics can constrain the solution space and reduce dependence on purely statistical correlations. Hybrid approaches that combine explicit physical models with learned representations may be particularly useful when complete analytical descriptions are unavailable but relevant prior knowledge exists. The relationship should also operate in both directions: physical knowledge can constrain learning, while data-driven models can compensate for aspects of experimental behavior that are difficult to describe analytically. The convergence of these approaches may provide a more reliable alternative than either purely model-based or purely data-driven reconstruction.

Physical fidelity must also be accompanied by uncertainty awareness. Most conventional ML outputs provide a prediction without directly communicating whether that prediction is reliable under the current experimental conditions. However, uncertainty may increase when images contain unfamiliar morphologies, low SNR, severe agglomeration, ambiguous boundaries, or acquisition conditions outside the training distribution. Uncertainty-aware approaches can identify these situations and distinguish high-confidence automated predictions from cases requiring additional measurements or expert review. Rather than being treated solely as an additional statistical output, uncertainty could therefore become an operational variable in future microscopy workflows.

This possibility becomes particularly significant when AI is integrated with automated acquisition. An uncertainty-aware system could identify regions for which additional information is required and respond by requesting higher-resolution imaging, modifying acquisition parameters, acquiring complementary spectroscopy, or flagging the result for human evaluation. Such feedback would transform AI from a passive post-processing tool into an active component of the experimental workflow. In this context, the objective of autonomous microscopy should not be the complete elimination of expert involvement, but the more effective allocation of expertise to measurements and interpretations for which computational confidence is insufficient.

An emerging extension of autonomous characterization is the integration of scientific reasoning into experimental decision-making. Kalinin et al. [94] discussed the transition from automated measurements toward knowledge-guided experimentation in electron and scanning probe microscopy, while Kandel et al. [95] experimentally demonstrated an AI-driven scanning workflow capable of selecting informative measurements during acquisition. More recently, Jamali et al. [96] proposed agentic electron microscopy frameworks in which specialized AI systems could contribute to experimental planning, interpretation of observations, and hypothesis generation. These developments suggest a progression from automated data acquisition and analysis toward AI-assisted scientific reasoning. Nevertheless, autonomous measurement selection should be distinguished from independent scientific interpretation, which remains an emerging research objective requiring experimental validation, physical consistency, and human oversight.

Another major research direction is the transition from isolated models toward integrated and multimodal characterization systems. Many current studies optimize individual tasks—such as denoising, segmentation, classification, or reconstruction—independently. However, real microscopy workflows are sequential and interconnected. Restoration can alter the information available for segmentation; segmentation determines the features used for quantitative measurement; and errors introduced at either stage may propagate into subsequent classification or materials-property interpretation. Evaluating individual models without considering this propagation may therefore overestimate their practical utility.

Future systems should increasingly integrate complementary architectures and learning paradigms according to the analytical objective. CNNs and residual networks can support feature extraction and restoration, encoder–decoder architectures can provide pixel-level segmentation, generative models can contribute to reconstruction, transformers can model longer-range spatial relationships, self-supervised approaches can exploit unlabeled data, and physics-informed learning can impose experimental constraints. The methodological challenge is not to identify a single architecture that replaces all others, but to determine how these complementary capabilities can be combined while preserving traceability and avoiding error amplification across the workflow.

Multimodal learning further expands this concept. Modern electron microscopy can generate morphological, compositional, spectroscopic, crystallographic, and three-dimensional information through combinations of SEM, TEM/STEM, EDS, EELS, diffraction, and tomography. These measurements are frequently analyzed independently despite originating from the same material system. Integrating them computationally could enable joint representations connecting morphology, composition, crystallography, processing conditions, and material properties. As illustrated by the biomedical examples discussed in Section 5, multimodal AI may provide information that is not accessible from any individual data source. However, successful implementation will require methods capable of managing differences in spatial resolution, acquisition geometry, dimensionality, noise characteristics, and uncertainty across modalities.

Three-dimensional characterization represents a particularly demanding example of this integration. Tomographic datasets require substantial acquisition time, storage, computational memory, alignment, reconstruction, segmentation, and quantitative analysis. High-resolution and spectroscopic 3D datasets further increase these requirements. Learning-based denoising and reconstruction may reduce the number of projections or electron dose required to obtain useful volumes [26,27], but computational cost remains substantial. Efficient architectures, model compression, optimized inference, distributed computing, and hardware-aware implementations will therefore be important for translating advanced models from specialized computing environments to routine microscopy laboratories. Computational efficiency should ultimately be evaluated not only by model-training time but also by its effect on the complete characterization workflow, including acquisition time, processing latency, storage requirements, and operator involvement.

Standardization and benchmarking remain equally important. The absence of widely adopted reference datasets, reporting practices, and evaluation protocols makes objective comparison among methods difficult. Studies may use different datasets, noise models, train–test splits, acquisition conditions, or evaluation metrics, meaning that apparently superior numerical performance may not indicate a genuinely more robust method. As discussed in Section 3, PSNR and SSIM provide useful but incomplete measures of restoration performance, while Dice, IoU, precision, recall, and related metrics characterize segmentation from different perspectives. None of these metrics alone establishes that the output preserves the physical quantities required for materials characterization.

Future benchmarking should therefore become increasingly task-oriented and physically meaningful. A denoising method intended for particle analysis, for example, should be evaluated not only according to PSNR or SSIM but also according to whether particle boundaries, size distributions, and morphology are preserved. A segmentation algorithm used for phase-fraction measurements should be evaluated according to the accuracy and uncertainty of the resulting quantitative measurement. Similarly, 3D reconstruction should be assessed according to structural fidelity and the reliability of volumetric quantities rather than visual plausibility alone. Shared datasets containing representative noise levels, low-dose conditions, complex morphologies, multiple material systems, and acquisition metadata would significantly improve reproducibility and enable more meaningful comparison of methods [13,26].

An additional challenge concerns the accuracy, reproducibility, and efficiency of quantitative measurements obtained through manual and AI-assisted electron microscopy analysis. Manual particle identification, boundary delineation, and defect quantification may be influenced by operator experience and interpretation criteria, introducing variability between observers. Rettenberger et al. [53] demonstrated that an uncertainty-aware Mask R-CNN model produced particle-size distributions broadly comparable to those obtained by three domain experts when analyzing SEM images of inorganic powders. For a dataset of 288 SEM images of LiCoO_2_, automated segmentation required approximately 3 min, compared with an average of 265 min for expert annotation. However, the model also missed some smaller particles, and substantial differences were observed among the human annotations, illustrating that agreement with expert measurements does not necessarily establish absolute accuracy. Jacobs et al. [97] investigated deep-learning-based segmentation of irradiation-induced defects in TEM images of FeCrAl alloys, evaluating defect morphology, size, and areal density rather than relying exclusively on segmentation performance metrics. Their analysis also highlighted the influence of expert-generated annotations and dataset characteristics on model reliability. Conversely, Nosi et al. [98] reported no statistically significant correlation between manual and machine-learning-based measurements of mitochondrial cristae length relative to mitochondrial surface area in a comparative TEM morphometry study, highlighting limitations in the automated recognition of complex ultrastructural features. Collectively, these findings indicate that AI-assisted analysis can substantially improve processing efficiency and support more consistent workflows, but its quantitative reliability remains dependent on specimen characteristics, annotation quality, segmentation accuracy, and the measured parameter. Therefore, automated measurements should be validated against appropriate reference data, inter-observer variability, and task-specific quantitative criteria before being used for scientific interpretation.

These considerations also suggest that future benchmarking should evaluate the complete computational pipeline rather than isolated model outputs. A method that produces modest improvement in image-quality metrics but substantially improves downstream quantitative measurements may be more scientifically valuable than one that maximizes PSNR without preserving critical boundaries. Conversely, a highly accurate segmentation model may have limited practical value if its performance collapses under modest changes in acquisition conditions. Benchmarking should therefore include robustness, transferability, uncertainty calibration, physical consistency, and downstream quantitative accuracy in addition to conventional image-based metrics.

The cross-disciplinary comparison with medical and biological imaging provides useful guidance for addressing these challenges. Biomedical AI has demonstrated the importance of external validation, heterogeneous datasets, uncertainty assessment, multimodal integration, and explicit evaluation of model generalization. Electron microscopy can adopt these methodological principles without assuming equivalence between the underlying imaging systems. In particular, the biomedical experience illustrates that increasingly complex models do not automatically produce increasingly trustworthy systems; reliability emerges from the interaction among data quality, model design, domain knowledge, validation strategy, and the context in which predictions are used.

On this basis, the future of AI-assisted electron microscopy can be understood as a transition across several interconnected dimensions: from large datasets to representative and traceable datasets; from internal accuracy to external generalization; from visually plausible outputs to physically consistent and uncertainty-aware predictions; from isolated algorithms to integrated multimodal workflows; and from model-centered metrics to task-oriented scientific validation. Progress along these dimensions is likely to be more consequential than improvements in architecture complexity alone.

Ultimately, the most promising direction is the development of AI systems in which data-driven learning, microscopy physics, multimodal information, uncertainty quantification, and experimental validation operate as complementary components of the same characterization framework. Such systems could reduce electron dose and acquisition time, improve reproducibility, automate repetitive analytical tasks, identify conditions requiring expert intervention, and connect structural and compositional measurements more effectively. Rather than replacing the microscopist, AI would increasingly function as an adaptive computational layer linking acquisition, reconstruction, interpretation, and experimental decision-making.

This perspective defines a broader research agenda for the field. The central challenge is no longer simply whether AI can improve an SEM or TEM image, segment a microstructure, or classify a feature—numerous studies have already demonstrated these capabilities. The more consequential question is whether AI can perform these tasks reliably across experimental domains, consistently with the underlying physics, transparently with respect to uncertainty, and in a form that improves the reproducibility and scientific value of the complete characterization workflow. Addressing this question will determine whether learning-based electron microscopy remains predominantly a collection of high-performing task-specific demonstrations or evolves toward robust, generalizable, and increasingly autonomous materials-characterization systems.

## 7. Conclusions

The evolution of image analysis in electron microscopy reflects a broader transition from deterministic, task-specific processing toward increasingly integrated, data-driven, and physically informed computational characterization. This review has examined that transition from the fundamental mechanisms governing SEM and TEM image formation and conventional filtering, restoration, and segmentation methods to classical machine learning (ML), deep learning (DL), self-supervised learning, generative approaches, transformers, foundation models, physics-informed learning, and multimodal analysis. Taken together, the evidence indicates that artificial intelligence (AI) is no longer limited to improving individual microscopy images; it is progressively becoming a computational framework capable of connecting acquisition, restoration, segmentation, reconstruction, quantitative analysis, and scientific interpretation.

Traditional image-processing methods remain valuable because of their interpretability, computational efficiency, and direct relationship with established signal-processing principles. Filtering, deconvolution, thresholding, and morphological operations can provide reliable results when noise characteristics, image-formation conditions, and microstructural features are sufficiently well defined. Their principal limitation emerges as experimental complexity increases: fixed mathematical assumptions and manually optimized parameters become progressively less transferable across heterogeneous microstructures, imaging conditions, instruments, and material systems. Learning-based approaches address part of this limitation by learning representations directly from data and modeling relationships that are difficult to encode explicitly. However, increased model flexibility does not automatically imply increased scientific reliability.

A central conclusion of this review is therefore that the transition from conventional image processing to AI should not be interpreted as the replacement of one family of algorithms by another. The strengths of traditional methods—including interpretability, explicit assumptions, and transparent validation—remain essential requirements for trustworthy AI-assisted microscopy. Conversely, ML and DL provide adaptability, contextual representation, and scalability that become increasingly important as microscopy datasets grow in size, dimensionality, and complexity. The most promising direction is therefore the integration of data-driven adaptability with physical knowledge, rigorous validation, and quantitative scientific objectives.

This perspective also changes how AI performance should be evaluated in electron microscopy. Improvements in PSNR, SSIM, segmentation accuracy, or visual image quality are useful but insufficient indicators of scientific performance. Restoration, segmentation, and reconstruction algorithms ultimately modify or interpret data from which physical quantities are extracted. A computational method should therefore be evaluated according to whether it preserves particle boundaries, morphology, phase distributions, defects, compositional information, volumetric structure, or other quantities relevant to the intended materials-characterization task. The appropriate question is consequently not only whether AI produces a better image, but whether it produces more reliable scientific information.

The comparison with medical and biological imaging provides an additional and distinctive perspective on this transition. Radiology, ultrasound, biomedical spectroscopy, and other biological imaging applications have confronted several challenges that are now increasingly relevant to AI-assisted electron microscopy, including limited and heterogeneous datasets, acquisition-dependent domain shifts, operator variability, three-dimensional reconstruction, multimodal data integration, uncertainty, and the need for external validation. The value of this comparison does not lie in assuming equivalence between biomedical and electron microscopy, which are governed by fundamentally different imaging physics and analytical objectives, but in identifying methodological insights that can be adapted across disciplines.

Radiological AI demonstrates particularly clearly that excellent internal performance does not guarantee generalization to new instruments, institutions, acquisition protocols, or populations. The corresponding challenge in electron microscopy is generalization across microscopes, detectors, acquisition parameters, laboratories, and material classes. Ultrasound provides complementary implications regarding operator-dependent acquisition and the integration of learning with image-formation physics, particularly for 3D reconstruction. Biomedical spectroscopy demonstrates how ML can extract information from complex, high-dimensional signals and how complementary imaging and molecular information can be combined into multimodal representations. Collectively, these experiences suggest that electron microscopy can benefit from methodological advances already being developed in biomedical AI, particularly external validation, physics-guided learning, uncertainty quantification, data-efficient training, and multimodal integration.

The cross-disciplinary analysis also reveals that the transfer of AI between fields should occur primarily at the level of methodological principles rather than pretrained algorithms alone. A network developed for a medical image cannot be assumed to represent electron-microscopy physics, just as a reconstruction strategy developed for ultrasound cannot be directly applied to electron tomography without accounting for differences in acquisition geometry and signal formation. Transferability therefore requires adaptation to electron–matter interactions, microscopy-specific noise statistics, structural scales, detector characteristics, and the physical quantities being measured. This distinction is critical for avoiding the assumption that increasing access to general-purpose or foundation models automatically resolves microscopy-specific analytical challenges.

Based on the evidence examined throughout this review, five interconnected requirements emerge for the next generation of AI-assisted electron microscopy: representative and traceable data, generalization beyond the training domain, physical consistency, multimodal integration, and uncertainty-aware validation. These requirements should not be addressed independently. Dataset diversity influences generalization; physical knowledge constrains reconstruction; multimodal information can reduce ambiguity; and uncertainty provides a mechanism for identifying conditions in which automated predictions should not be accepted without additional evidence. Their integration therefore provides a more meaningful framework for future development than the isolated pursuit of increasingly complex architectures.

This integration also provides a pathway toward more autonomous microscopy. Future systems may combine automated acquisition with restoration, feature detection, segmentation, spectroscopy, 3D reconstruction, and quantitative analysis while continuously evaluating prediction confidence. Rather than simply processing images after acquisition, AI could help determine where additional information is required, identify regions of interest, recommend complementary measurements, or flag uncertain results for expert evaluation. In such workflows, autonomy should not be understood as removal of the researcher from the characterization process, but as a redistribution of tasks in which repetitive and data-intensive operations are increasingly automated while scientific expertise remains central to experimental design, validation, and interpretation.

Accordingly, the long-term impact of AI in electron microscopy is likely to extend beyond image enhancement. Its greater potential lies in connecting previously separated stages of materials characterization and integrating structural, compositional, spectroscopic, crystallographic, and three-dimensional information into coherent computational representations. Such integration could strengthen relationships among processing conditions, microstructure, composition, and material properties while reducing analysis time and operator dependence. It could also facilitate high-throughput characterization strategies in which increasingly large datasets are analyzed with greater consistency and traceability.

At the same time, substantial challenges remain before this vision can be realized. Standardized datasets and benchmarking protocols are still limited; external validation across instruments and material systems remains uncommon; computational requirements can restrict deployment of advanced models; and physically plausible outputs are not necessarily physically correct. Addressing these limitations will require closer interaction among microscopists, materials scientists, computer scientists, instrument developers, and researchers working in adjacent imaging disciplines. The biomedical experience discussed in this review demonstrates that technological sophistication must be accompanied by equally rigorous attention to validation, reproducibility, uncertainty, and domain-specific interpretation.

Ultimately, the principal advance enabled by AI in electron microscopy should not be measured by the complexity of the model or by its ability to generate visually improved images, but by its capacity to produce reproducible, physically meaningful, and quantitatively reliable materials information. The convergence of conventional image-processing principles, data-efficient learning, physics-informed AI, multimodal analysis, uncertainty quantification, and external validation provides a pathway toward this objective. By combining these developments with methodological findings from medical and biological imaging, electron microscopy can progress from isolated AI-assisted tasks toward robust, transferable, and increasingly autonomous characterization workflows. This transition has the potential to redefine the role of computational analysis in SEM and TEM, transforming AI from a post-processing tool into an integral component of experimental materials characterization.

## Figures and Tables

**Table 1:** Comparative summary of conventional image-processing and deep-learning-based techniques for SEM and TEM images.

Technique	Typical PSNR Trend	Typical SSIM Trend	Reference	Strengths	Limitations
Gaussian Filtering	Moderate	Low–Moderate	[25,26]	Fast implementation and effective noise reduction	Blurring of edges and loss of fine structural details
Median Filtering	Moderate	Moderate	[25,26]	Better edge preservation than linear filters	May distort texture information
Wiener Deconvolution	Moderate–High	Moderate	[8]	Effective blur reduction when PSF is known	Highly sensitive to PSF estimation
Richardson–Lucy Deconvolution	High	Moderate–High	[8]	Enhanced image sharpness and detail recovery	Amplification of noise and ringing artifacts
Contrast-Limited Adaptive Histogram Equalization (CLAHE)	Variable	Moderate	[25]	Significant local contrast enhancement	May amplify image noise
CNN-Based Enhancement	High	High	[8,30,31,32,33]	Improved feature preservation and robustness	Requires training datasets and computational resources
GAN-Based Reconstruction	Very High	Very High	[33,34,35]	Superior image reconstruction and super-resolution capabilities	High computational cost and training complexity

Abbreviations: CLAHE, contrast-limited adaptive histogram equalization; CNN, convolutional neural network; GAN, generative adversarial network; PSNR, peak signal-to-noise ratio; SSIM, structural similarity index measure; PSF, point spread function. The reported PSNR and SSIM trends are qualitative and depend on the dataset, acquisition conditions, implementation parameters, and evaluation protocol; they should not be interpreted as universal performance rankings.

**Table 2:** Representative families of classical supervised machine learning algorithms applied to electron microscopy image analysis.

ML Family	Representative Algorithms	Typical Applications	Advantages	Limitations	Foundational Reference	Representative SEM/TEM Application
Margin-based learning	SVM	Mineral classification	High accuracy with small datasets	Sensitive to feature engineering	[36]	[37]
Tree-based learning	DT, RF	SEM–EDS phase classification	Robust and interpretable	Limited feature representation	[38]	[37]
Instance-based learning	k-NN	Particle recognition	Simple and effective	High computational cost	[39]	[9]
Linear probabilistic models	Logistic Regression	Binary classification	Fast and interpretable	Limited nonlinear modeling	[40]	[9]
Artificial Neural networks	Shallow ANN	Morphology recognition	Nonlinear learning capability	Lower representational power than DL	[41]	[10,29]

**Table 3:** Representative applications of machine learning in biomedical imaging and materials characterization. The table presents selected applications and supporting references, highlighting methodological parallels between biomedical data analysis and electron microscopy without implying direct model transfer between modalities.

Key References	Typical Tasks	Machine Learning Application	Imaging Modality	Field
[71,73]	Disease detection, prognosis prediction	Deep learning classification	CT, X-ray, MRI	Radiology
[72]	Tumor detection, tissue classification	CNN-based segmentation and detection	Sonography	Ultrasound
[82]	Molecular diagnostics, disease detection	ML spectral classification	Raman spectroscopy	Biomedical spectroscopy
[75,77]	Disease monitoring and outbreak prediction	Predictive modeling	Clinical and epidemiological data (non-imaging)	COVID-19 management
[10,13]	Particle detection, morphology analysis	Deep learning image analysis	SEM	Electron microscopy
[3,34]	Mineral classification and elemental mapping/tomography	ML-based mineral classification; complementary spectroscopic tomography	SEM-EDS/TEM-EELS	Multimodal microscopy

Note: The applications listed in Table 3 represent distinct examples from biomedical imaging and electron microscopy. Similarities in machine learning tasks or architectures indicate methodological relevance but do not establish direct transfer of pretrained models or equivalent performance across modalities. The supporting references should be interpreted within their respective experimental contexts.

## Data Availability

No new data were created or analyzed in this study. Data sharing is not applicable to this article.
